# Engineered Foxp1^high^ Exosomes Ameliorates Systemic Lupus Erythematosus

**DOI:** 10.1002/advs.202415712

**Published:** 2025-07-03

**Authors:** Luhan Niu, Qianmin Ou, Qianhui Ren, Zhengshi Li, Hongcheng Chen, Fangcao Lei, Xueli Mao, Songtao Shi, Zetao Chen, Wei Teng

**Affiliations:** ^1^ South China Center of Craniofacial Stem Cell Research Hospital of Stomatology Sun Yat‐sen University Guangdong Provincial Key Laboratory of Stomatology Guangzhou 510080 China; ^2^ Center for Stem Cell Biology and Tissue Engineering Key Laboratory for Stem Cells and Tissue Engineering Ministry of Education Sun Yat‐Sen University Guangzhou 510080 China

**Keywords:** aggregation, exosome engineering, foxp1, regulatory T cells, systemic lupus erythematosus

## Abstract

Systemic lupus erythematosus (SLE) is an autoimmune disease characterized by immune dysregulation and impaired Treg cell differentiation. Mesenchymal stem cell‐derived exosomes (MSC‐exos) hold promise for treating immune‐related diseases, while their clinical application is hindered by the limited production and non‐specific organ distribution. In this study, a combined engineering strategy is developed for MSC‐exo via aggregation culture and genetic editing, achieving a substantial increase in both exosome yield and therapeutic specificity in SLE. First, MSCs produce a high yield of engineered exosomes through an aggregation culture engineering strategy (Agg‐exo), demonstrating immune organ targeting and promoting Tregs via the Foxp1/STAT5/Foxp3 axis. Then, MSCs are engineered by overexpressing Foxp1 in order to acquire Foxp1^high^ Agg‐exo with enhanced immunomodulatory properties, which showes superior therapeutic effect for SLE. Taken together, a newly dual‐engineering strategy is developed to produce high‐yield, Foxp1^high^ Agg‐exo, which solved the limitation of low‐yield production and non‐specific organ distribution of MSC‐exos. This innovative strategy holds great potential for the development of exosome‐based therapies in autoimmune diseases.

## Graphical abstract

This study presents a dual‐engineering strategy combining mesenchymal stem cell aggregation and Foxp1 overexpression to generate Foxp1^high^ aggregation exosomes with enhanced immunomodulatory properties. These engineered exosomes exhibit immune organ targeting and superior efficacy in systemic lupus erythematosus treatment by promoting Treg differentiation via the Foxp1/STAT5/Foxp3 axis, offering a novel approach for autoimmune disease therapy.

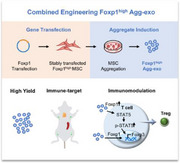

## 1 Introduction

Systemic lupus erythematosus (SLE) is an autoimmune disease marked by immune dysregulation, particularly involving a deficiency in regulatory T cells (Tregs), which are crucial for maintaining immune self‐tolerance. The immune imbalance contributes to disease activity and organ damage in SLE, and currently, no treatment can fully reset the immune system to achieve drug‐free remission.^[^ ^1^, ^2^, ^3^ ^]^ Mesenchymal stem cells (MSCs) are versatile multipotent cells known for their immunomodulatory capabilities, making them a promising candidate for treating autoimmune diseases such as SLE.^[^ ^4^ ^]^ Recent attention has focused on MSC‐derived exosomes (MSC‐exos), small extracellular vesicles that encapsulate bioactive molecules, including DNA, RNA, and proteins, which show promising immunomodulatory capabilities and low cytotoxicity.^[^ ^5^, ^6^, ^7^, ^8^, ^9^ ^]^ Despite their promise, MSC‐exos face significant challenges that hinder their clinical application: 1) Limited production, the low yields under standard culture conditions, making large‐scale production difficult.^[^ ^10^ ^]^ 2) Poor organ targeting, with broad distribution to non‐target organs such as the liver and lungs, resulting in insufficient treatment specificity.^[^ ^11^, ^12^, ^13^ ^]^

Numerous engineering strategies for extracellular vesicles are being explored to address the challenges limiting the clinical application of exosomes, including improving their stability, bioactivity, and targeting efficiency.^[^ ^14^, ^15^, ^16^ ^]^ The engineering strategies are mainly categorized into lipid insertion, membrane fusion, nanoparticle encapsulation, and gene editing. Notably, modification of exosome components can be achieved by gene editing of parent cells or modulating culture conditions, such as aggregate inducing and chemically defined media.^[^ ^14^, ^17^, ^18^ ^]^ Additionally, lentiviral vectors are widely employed as gene editing ways to deliver transgenes into MSCs, enabling targeted protein overexpression that refines exosome function by tailoring their molecular cargo for specific therapeutic effects.^[^ ^19^, ^20^ ^]^ However, none of these engineering approaches alone addresses the limitations of exosome clinical applications well. Here, we developed a combined engineering strategy to enhance exosome yield using aggregation culture. Therapeutic specificity was further refined through targeted gene editing, presenting a promising approach for SLE treatment.

Abnormal differentiation of CD4^+^ T cells is a key factor in SLE. Pathogenic CD4^+^ T cells promote autoantibody production and drive the infiltration of affected organs, such as the kidneys and skin.^[^ ^21^ ^]^ Meanwhile, Tregs are deficient in both number and function, and the imbalance between Th17 cells and Tregs is a hallmark of disease activity. Foxp3 is the master transcription factor of Tregs for maintaining their suppressive function through its constitutive expression.^[^ ^22^ ^]^ Forkhead box protein P1 (Foxp1) is ubiquitously expressed and implicated in both physiological and pathological processes, such as T‐cell quiescence, T‐cell exhaustion, antitumor immunity, and CD4^+^ T‐cell differentiation.^[^ ^23^ ^]^ Foxp1 can bind to the Foxp3 locus to stabilize Foxp3 expression, further preserve the integrity of the Foxp3 locus, and regulate Foxp3 chromatin binding.^[^ ^24^, ^25^, ^26^ ^]^ Therefore, Foxp1 has the potential to influence Treg differentiation and function, but the underlying mechanisms remain unclear.

Here, we use a dual engineering strategy combined with aggregation culture and gene editing to generate Foxp1^high^ Agg‐exo, with high yield, innate immune organ‐targeting, and potent immunoregulation capacity for SLE. These combined engineered exosomes provide a promising therapeutic avenue for targeted exosome‐based treatment of SLE and other autoimmune diseases.

## 2 Results

### 2.1 Characterization of Engineered Aggregated MSC‐Derived Exosomes

**Figure 1:**
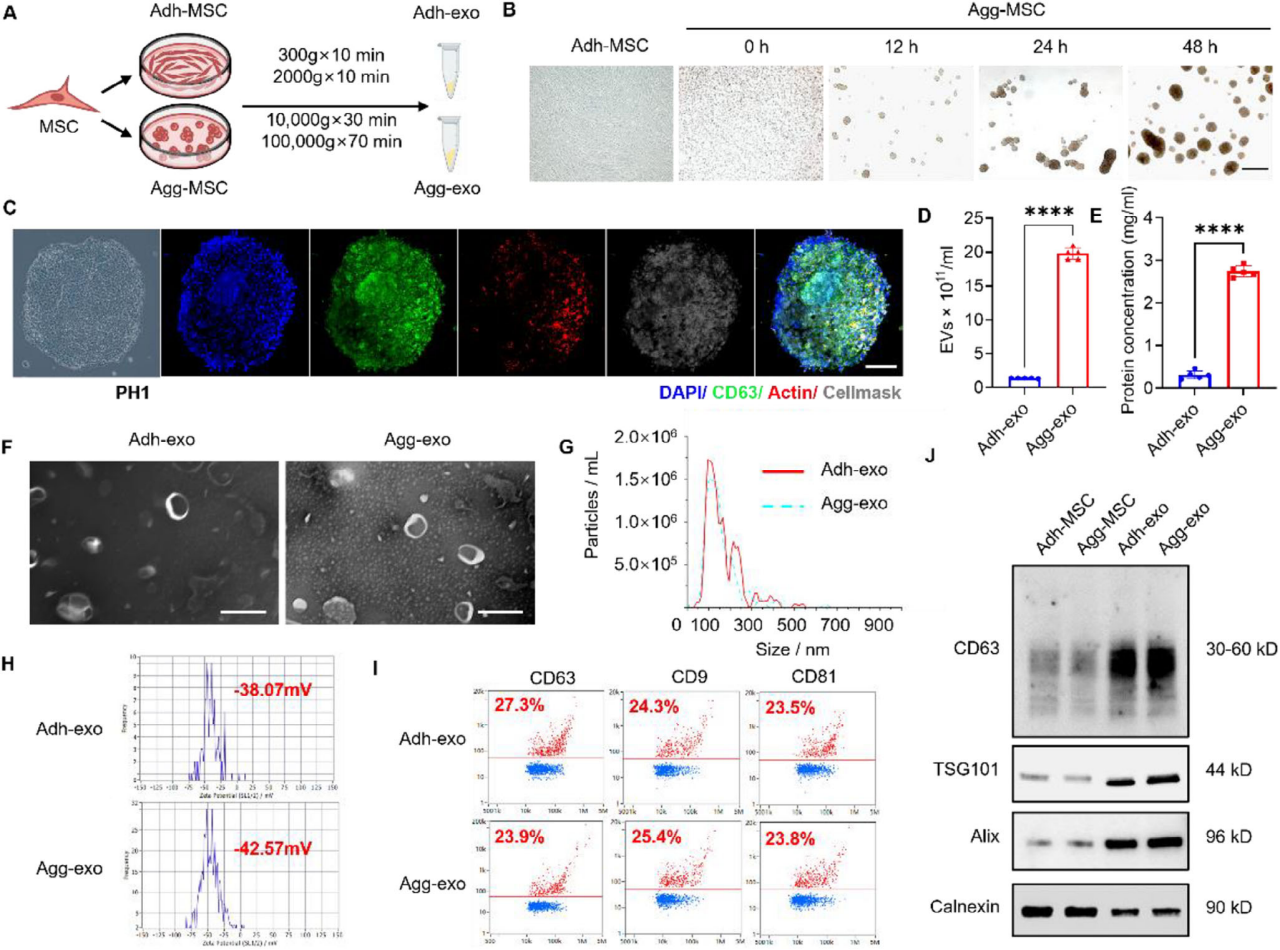
Suspension culture system induces MSC aggregation and engineered exosome characterization. A) Schematic representation of MSC culture as well as exosome isolation methods. B) Under the light microscope, Adh‐MSCs were observed to have a shuttle‐shaped, swirling arrangement; whereas Agg‐MSCs clusters started to appear after 12 h of culture, and stable, dense cell aggregates were formed at 48 h, scale bar, 200 µm. C) MSC aggregates cultured for 48 h in low adhesion were seen as dense structures under PH1 phase contrast microscopy; immunofluorescence results showed extensive expression of CD63, scale bar, 100 µm. D) The nanoparticle tracking analysis (NTA) results showed that equivalent MSC produced much higher amounts of Agg‐exo than Adh‐exo, *n* = 5. E) The Pierce BCA Protein Assay. Equal amounts of MSC produced much higher levels of Agg‐exo protein than Adh‐exo, *n* = 5. F) TEM showed that the isolated Adh‐exo and Agg‐exo presented an oval shape and a typical cup‐shaped structure, scale bar, 200 nm. G,H) NTA particle size analysis showed that the isolated Adh‐exo and Agg‐exo particle sizes were concentrated at <150 nm, and the average potentials of both were −38.07 and −42.57 mV, respectively. I) NanoFCM results showed that both Adh‐exo and Agg‐exo highly expressed exosome surface markers:3 CD63, CD9, and CD81. J) The western blot results showed that both Adh‐exo and Agg‐exo expressed high levels of CD63, TSG101, and Alix, and low levels of calnexin. ns, not significant, ^*^ *p* < 0.05; ^**^ *p* < 0.01; ^***^ *p* < 0.001; ^****^ *p* < 0.0001.

We cultured equal numbers of MSCs under suspension conditions and traditional adherent conditions, respectively. Then, the exosomes were isolated from the culture media using differential centrifugation (**Figure** **1**A). MSCs formed multicellular spheroids in suspension culture, contrasting with the spindle‐shaped and vortex‐like arrangement observed in adherent culture (Figure 1B).^[^ ^27^ ^]^ Immunofluorescence staining of the MSCs' spheroids revealed tight cell‐cell adhesion and elevated expression of CD63, a pivotal exosome marker (Figure 1C).^[^ ^28^ ^]^

Exosomes were collected from equal volumes of MSCs supernatants in both culture conditions and quantified using nanoparticle tracking analysis (NTA) and bicinchoninic acid assay (BCA). Results indicated that the exosome yield and protein concentration were significantly higher in Agg‐exo than in Adh‐exo (Figure 1D,E). Transmission electron microscopy (TEM) and NTA further characterized the exosomes, showing both Agg‐exo and Adh‐exo exhibited typical cup‐shaped vesicles, predominantly <150 nm in size, with the average zeta potentials of −38.07 and −42.57 mV, respectively (Figure 1F–H).^[^ ^29^ ^]^ Nano‐flow cytometry confirmed positive expression of exosome makers CD63, CD9, and CD81 in both exosome types (Figure 1I). Western blot analysis demonstrated high expression of transmembrane protein CD63, along with cytoplasmic protein TSG101 and Alix, and minimal expression of calnexin in both Agg‐exo and Adh‐exo (Figure 1J). These data indicate that we successfully isolated high‐yield Agg‐exos through suspension‐induced aggregation culture.

### 2.2 Systemic Infusion of Engineered Agg‐Exo Shows Improvement in Ameliorating SLE

**Figure 2:**
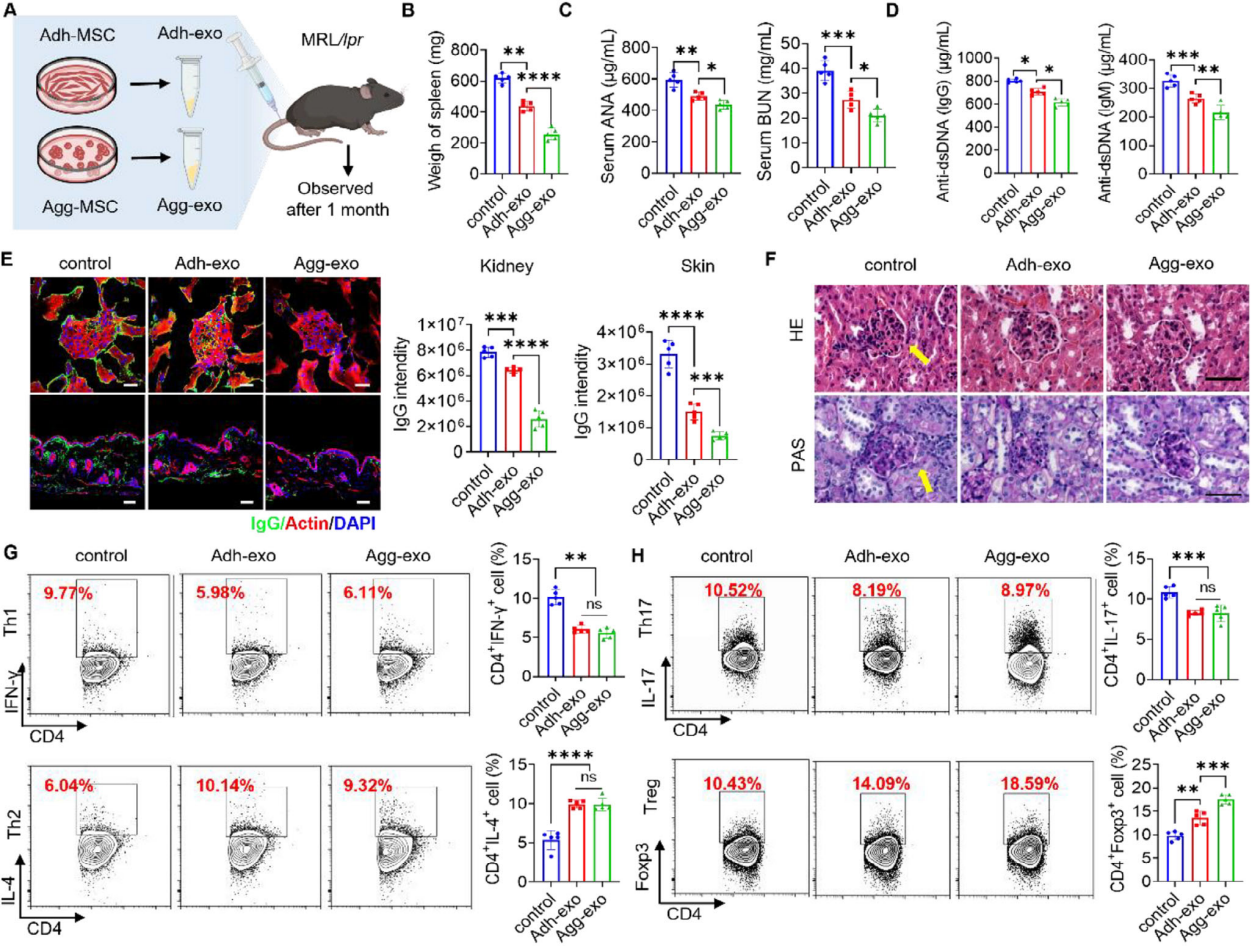
Agg‐exos exhibit a better therapeutic effect on SLE. A) Schematic diagram showing our exosome treatment protocol for MRL/*lpr* mice and efficacy assessment. B) In the spleen weight of MRL/*lpr*, Agg‐exo reduced the weight of the spleen more than the Adh‐exo group, compared to the control group, *n* = 5. C,D) The concentration of serum ANA, BUN, and dsDNA (IgG) in the Agg‐exo declined more than the Adh‐exo group, compared to the control group, while dsDNA (IgG) did not differ between the two treatment components, *n* = 5. E) Confocal assay confirmed the IgG less deposit in the kidney and skin of under exosome treatment, with significantly reduced effect in the Agg‐exo group, *n* = 5; scale bar, 20 µm for kidney; scale bar, 50 µm for skin. F) H&E and PAS staining results showed the diminished kidney damage under exosome treatment, while the basement membrane integrity and inflammatory cell infiltration were less in the Agg‐exo treated group, the arrow indicated the glomerular margin is broken, scale bar, 100 µm. G,H) Flow cytometry showed that the ratio of CD4^+^IFN‐γ^+^ Th1 cells and CD4^+^Foxp3^+^ Treg cells increased in the spleen of MRL/*lpr* mice, while the ratio of CD4^+^IL‐4^+^ Th2 cells and CD4^+^IL‐17^+^ Th17 cells decreased compared with control group, noticeably, the ratio of Treg cells under Agg‐exos significantly higher than Adh‐exo, while no difference in other cells, *n* = 5. ns, not significant, ^*^ *p* < 0.05; ^**^ *p* < 0.01; ^***^ *p* < 0.001; ^****^ *p* < 0.0001.

Previous studies have suggested that MSC‐exo effectively alleviates SLE symptoms primarily through their immunomodulatory properties.^[^ ^30^, ^31^ ^]^ The MRL/*lpr* mouse is a commonly used spontaneous mouse model of SLE with severe lupus nephritis.^[^ ^32^ ^]^ In this study, we systemically administered engineered Agg‐exo and Adh‐exo to female MRL/*lpr* mice (12–16 weeks old) via tail vein infusion, followed by pathological assessments at one‐month post‐treatment (**Figure** **2**A).

Results demonstrated that the Agg‐exo treatment group showed significantly lower spleen weights and decreased serum levels of ANA, BUN, and anti‐dsDNA, indicating an improved systemic disease state compared to both the control and Adh‐exo groups (Figure 2B–D). Additionally, IgG deposition in the spleen and skin was reduced compared to the Adh‐exo group, confirming a stronger effect in mitigating immune complex accumulation compared to Adh‐exo (Figure 2E). Histopathologic analysis of kidney sections stained with HE and PAS showed that Agg‐exo treatment more effectively preserved glomerular integrity, reduced mesangial cell proliferation, and mitigated basement membrane disruption (Figure 2F). Notably, immune cell profiling by flow cytometry showed that both exosome treatment groups exhibited a reduction in Th1 and Th17 cells, alongside an increase in Treg and Th2 cells (Figure 2G,H). However, the increase in Treg cells was significantly greater in the Agg‐exo group compared to Adh‐exo, highlighting the superior immunomodulatory effects of Agg‐exo in restoring immune homeostasis (Figure 2H). These data suggest that systemic infusion of engineered Agg‐exo provides significant therapeutic benefits in ameliorating SLE pathology in MRL/*lpr* mice, with effects superior to the Adh‐exo group.

### 2.3 Engineered Agg‐Exo Exhibit Immune‐Targeting After Systemic Infusion in MRL/lpr Mice

Following systemic administration, unmodified exosomes typically accumulate in the liver, kidneys, and spleen, with being rapidly eliminated and reach low concentrations in target tissues. However, exosomes derived from certain cell types can preferentially target immune cells, such as T cells, B cells, and macrophages. This targeting is attributed to the high expression of transmembrane proteins (LAMP‐2b, ICAM‐1) and naturally occurring molecules (EBV gp350, RVG).^[^ ^12^, ^33^, ^34^, ^35^ ^]^ The biodistribution of exosomes directly influences their therapeutic efficacy and potential toxicity.

**Figure 3:**
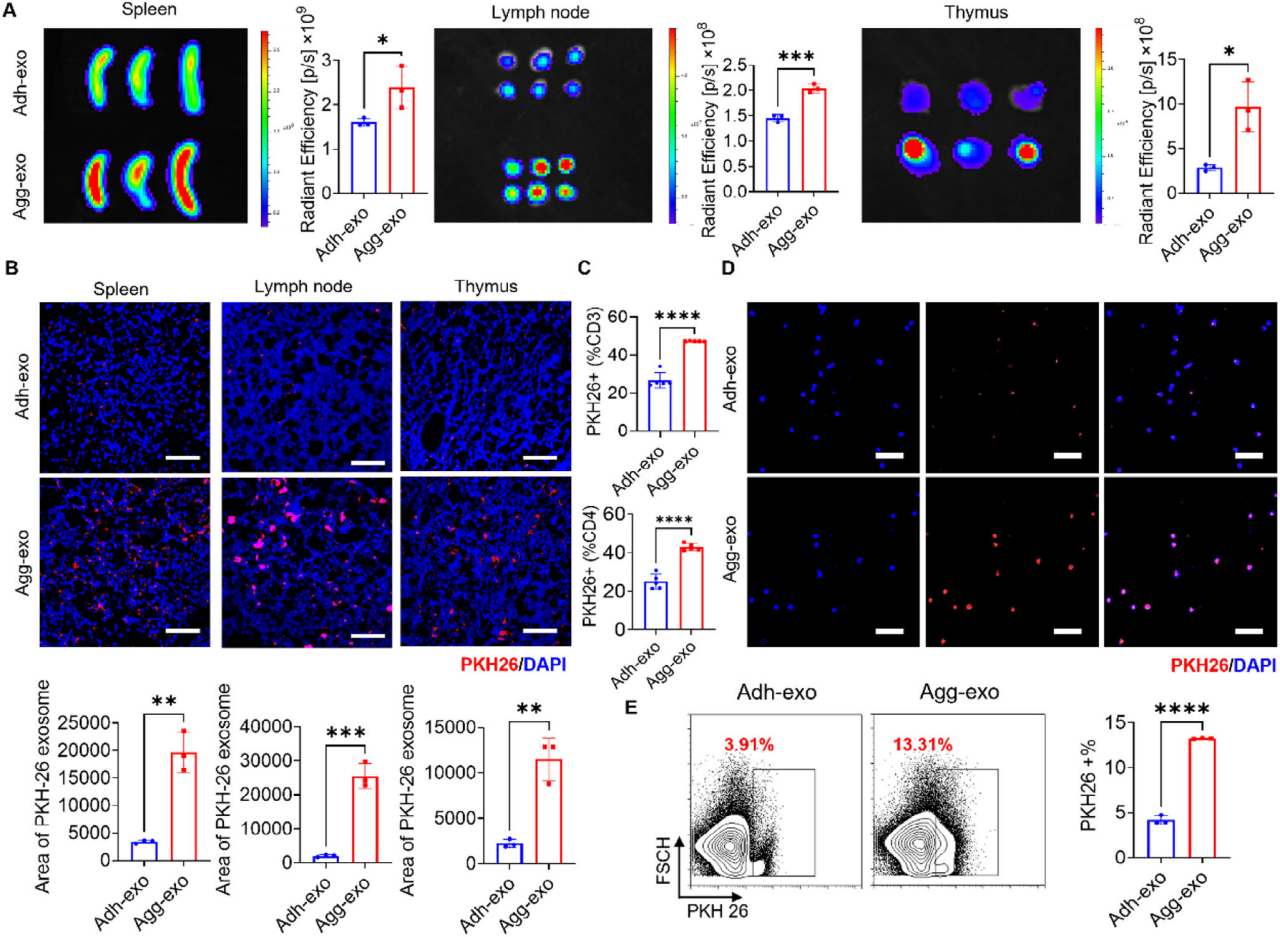
Agg‐exo presents an immune organ tendency in body distribution. A,B) IVIS analysis (A) and immunofluorescence staining of frozen tissue sections (B) showed that when DiR/PKH26‐labelled exosome was systemically infused into MRL/*lpr* mice for 24 h, the deposition of Agg‐exo in the spleen, lymph node, and thymus was much higher than that of Adh‐exo, *n* = 3, scale bar, 50 µm. C) Flow cytometry analysis shows that compared to Adh‐exo, Agg‐exo exhibits significantly higher uptake by T lymphocytes and CD4^+^ T helper cells in vivo, *n* = 5 D) Immunofluorescence results showed that PKH26 fluorescence co‐localized more with CD4^+^ T cells in the Agg‐exo group after 24 h of co‐culture of PKH‐26‐labelled exosome with CD4^+^ T cells in vitro, scale bar, 40 µm. E) Flow cytometry showed more cells expressing PKH26 fluorescence in the Agg‐exo group, after 24 h of co‐culture of PKH‐26‐labelled exosome with CD4^+^ T cells in vitro, *n* = 3. ns, not significant, ^*^ *p* < 0.05; ^**^ *p* < 0.01; ^***^ *p* < 0.001; ^****^ *p* < 0.0001.

To investigate the differential biodistribution of engineered Agg‐exo versus Adh‐exo in MRL/*lpr* mice post‐systemic infusion, we labeled exosomes with PKH‐26 and DiR Iodide probe, followed by tail vein injection and monitored 24 h later.^[^ ^36^ ^]^ In vivo imaging systems (IVIS) and fluorescence staining of tissue sections revealed that both Agg‐exos and Adh‐exos primarily concentrated in the liver, kidneys, and spleen, consistent with previous reports (**Figure** **3**A; Figure S1A, Supporting Information). However, Agg‐exo showed significantly greater enrichment in immune‐related organs, including spleen, lymph node, and thymus, compared to Adh‐exo (Figure 3A,B). No notable differences were observed in the distribution of exosomes in other organs (Figure S1A,B, Supporting Information).

Furthermore, flow cytometry analysis of splenic immune cells revealed that CD3⁺ T lymphocytes and CD4⁺ T helper cells exhibited significantly higher uptake of Agg‐exo compared to Adh‐exo, while neutrophils, B lymphocytes, and macrophages showed no significant differences in exosome capture (Figure 3C; Figure S1C, Supporting Information). Given the critical role of CD4^+^ T cells in SLE progression, we isolated splenic CD4^+^ T cells from MRL*/lpr* mice to further validate the immune‐targeted distribution of exosomes in vitro. After 24 h of co‐culture with PHK26‐labelled exosomes, both immunofluorescence staining and flow cytometry confirmed that Agg‐exo were more efficiently captured by CD4^+^ T cells than Adh‐exo (Figure 3D,E). These data demonstrate that systemically infused engineered Agg‐exo exhibit immune‐preferential biodistribution in MRL/*lpr* mice.

### 2.4 Engineered Agg‐Exo Promote Treg Differentiation in MRL/lpr Mice

**Figure 4:**
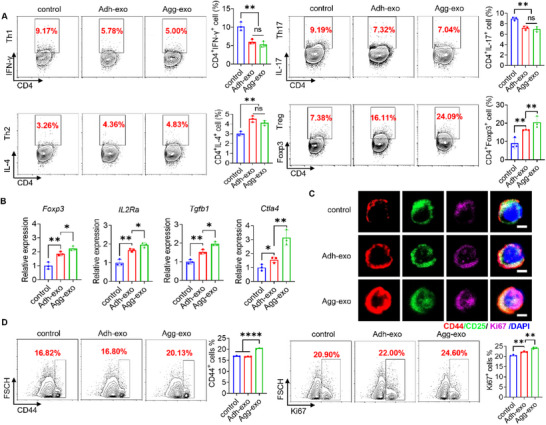
Agg‐exo regulates the differentiation of CD4^+^ T cells into Treg. A) Flow cytometry of CD4^+^ T cell, co‐cultured with exosome in vitro, showed that the ratio of CD4^+^IFN‐γ^+^ Th1 cells and CD4^+^Foxp3^+^ Treg cells increased in the spleen of MRL/*lpr* mice, while the ratio of CD4^+^IL‐4^+^ Th2 cells and CD4^+^IL‐17^+^ Th17 cells decreased compared with control group, noticeably, the ratio of Treg cells under Agg‐exos significantly higher than Adh‐exo, while no difference in other cells, *n* = 3. B) Treg‐related gene expression was assessed by qRT‐PCR. Agg‐exo treatment significantly increased the expression of *Foxp3, IL2Ra, Tgfb1, Ctla4*, *n* = 3. C) Immunofluorescence of CD4^+^ T cells showed that CD4^+^ T cells under Agg‐exo co‐culture highly expressed CD44, CD25, and Ki67, which are associated with Treg cell activity, scale bar, 2 µm. D) Flow cytometry of CD4^+^ T cells showed that CD4^+^ T cells under Agg‐exo co‐culture highly expressed CD44 and Ki67, n = 3. ns, not significant, ^*^ *p* < 0.05; ^**^ *p* < 0.01; ^***^ *p* < 0.001; ^****^ *p* < 0.0001.

T cell isotype disorder plays a central role in the pathogenesis of SLE, due to its association with MHC proteins, and it contributes to lupus‐like symptoms in MRL/*lpr* models.^[^ ^37^ ^]^ Tregs, essential for maintaining immune tolerance, are often identified as deficient or defective in both mouse models and human studies of SLE.^[^ ^37^ ^]^ Therefore, we further co‐cultured engineered Agg‐exo and Adh‐exo with splenic CD4^+^ T cells isolated from MRL/*lpr* mice. Both exosomes resulted in a decreased ratio of CD4^+^IFN‐γ^+^ Th1 and CD4^+^IL‐17^+^ Th17 cells, along with an increased ratio of CD4^+^IL‐4^+^ Th2 and CD4^+^Foxp3^+^ Treg cells (**Figure** **4**A). Notably, Agg‐exo led to a significant increase in the Treg population, consistent with previous in vivo observations of CD4^+^ T cells. Neither Agg‐exo nor Adh‐exo affected CD4^+^T cells' viability (Figure S2A, Supporting Information).

Subsequently, we performed Treg induction using CD4^+^ T cells in vitro, and flow cytometry analysis revealed a significant increase in CD4^+^Foxp3^+^ Tregs (Figure S2B, Supporting Information). The expression levels of Treg differentiation genes, including *Forkhead box protein P3 (Foxp3)*, *Interleukin 2 receptor subunit alpha (IL2Ra)*, *Transforming growth factor‐beta (Tgfb1)*, and *Cytotoxic T lymphocyte‐associated protein‐4 (Ctla4)*, were significantly higher in Agg‐exo co‐culture compared to Adh‐exo (Figure 4B). Additionally, we assessed Tregs' function by immunofluorescence and flow cytometry, focusing on the expression of CD44, CD25, and Ki67 as markers of Treg activity. These markers were more highly expressed under Agg‐exo co‐culture than Adh‐exo, indicating that Agg‐exo enhanced the Treg functional activity (Figure 4C,D; Figure S2C, Supporting Information). Treg suppression assay reveals that Agg‐exo‐treated Tregs exhibit significantly greater suppressive capacity on effector T cell (Teff) proliferation compared to other groups (Figure S2D, Supporting Information). Furthermore, Agg‐exo treatment in vivo can significantly increase the proportion of CD69^+^ T cells and CTLA‐4^+^ T cells in MRL/*lpr* mice, indicating enhanced T cell activation (Figure S2E, Supporting Information). These findings underscore the superior ability of engineered Agg‐exos to drive both the differentiation and functional activation of Tregs.

### 2.5 Engineered Agg‐Exos Promotes Tregs Differentiation via the Foxp1/STAT5/ Foxp3 Axis

Phosphorylation and nuclear translocation of STAT5, which further induces Foxp3 expression, is a critical step in Treg cell differentiation and immune suppressive function.^[^ ^38^, ^39^, ^40^ ^]^ Foxp1 is another member of the forkhead transcription factor family and a sibling of Foxp3. Although Foxp1 has not been definitively reported to regulate Treg differentiation, it is essential for maintaining optimal Foxp3 expression in Tregs.^[^ ^25^ ^]^ Additionally, Foxp1 has been shown to influence lymphoblastic leukemia and hippocampal neuron injury by modulating STAT family expression.^[^ ^41^, ^42^ ^]^

**Figure 5:**
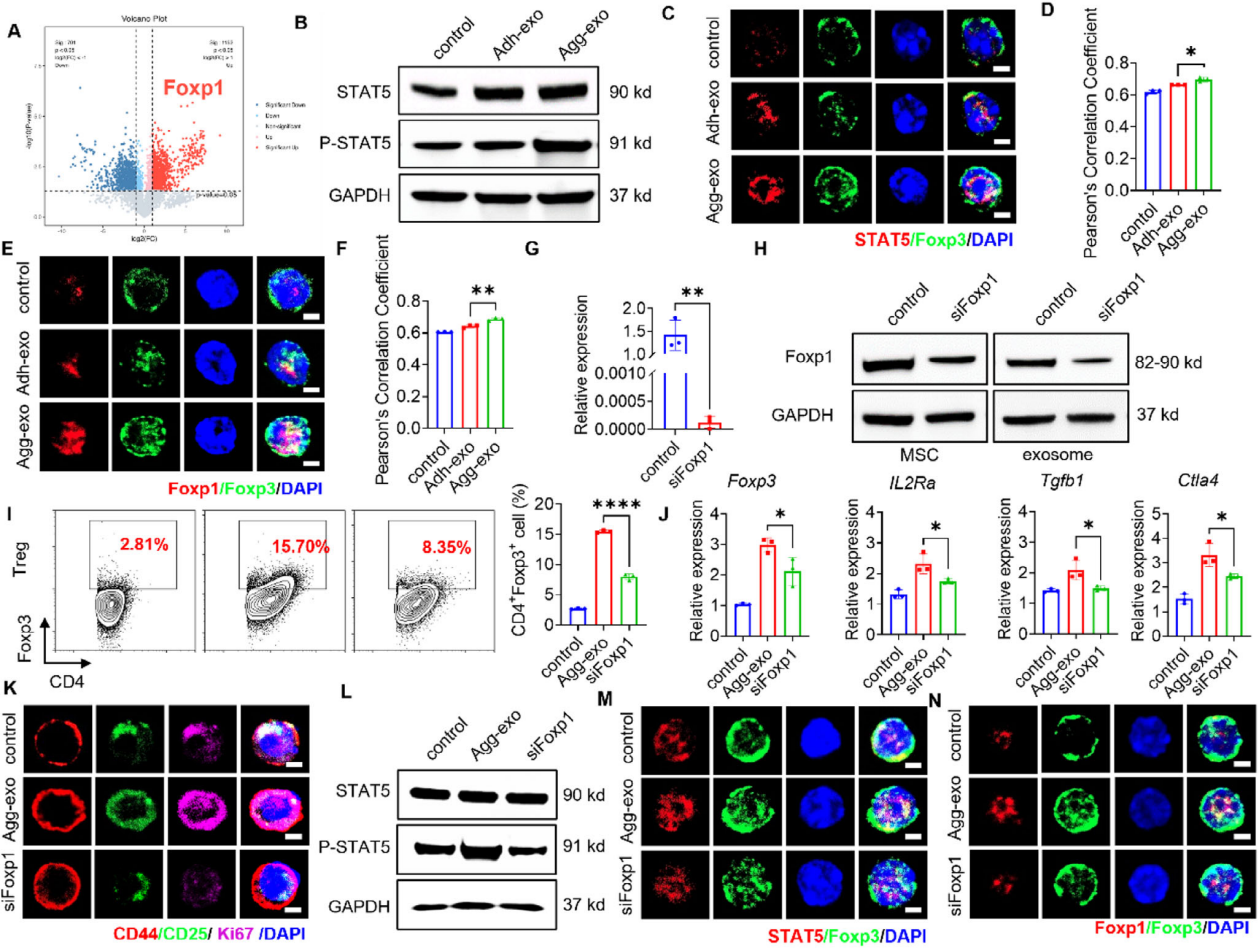
Agg‐exos regulates Treg differentiation via Foxp1/STAT5/Foxp3. A) Among the differentially proteins analyzed by proteomics, Agg‐exo significantly over‐expressed Foxp1. B) Western blot of CD4^+^ T cell, co‐cultured with exosomes, showed Agg‐exo group highly expressed p‐STAT5, while expression of STAT5 was not significantly different between groups. C,D) Immunofluorescence results showed significantly high expression and increased fluorescence co‐localization of STAT5 and Foxp3 in the nuclei of Agg‐exo‐treated CD4^+^ T cells, *n* = 3, scale bar,2 µm. E,F) Immunofluorescence results showed increased Foxp1 expression and increased fluorescence co‐localization with Foxp3 in the nuclei of Agg‐exo‐treated CD4^+^ T cells, *n* = 3, scale bar, 2 µm. G,H) We used RNAi to silence Foxp1 expressed in MSCs, which were assessed by qRT‐PCR and western blot. The *Foxp1* gene expression significantly decreased in siFoxp1 MSCs (G), and Foxp1 was lowly expressed in siFoxp1 MSCs, as well as siFoxp1 Agg‐exo (H), *n* = 3. I) Flow cytometry showed siFoxp1 Agg‐exo induced a significantly lower percentage of Treg differentiation compared to Agg‐exo, *n* = 3. J) Treg‐related gene expression was assessed by qRT‐PCR. The siFoxp1 Agg‐exo group induced a decrease in the expression of these genes (Foxp3, IL2Ra, Tgfb1, Ctla4), compared to Agg‐exo, *n* = 3. K) Immunofluorescence of CD4^+^ T cells showed that CD4^+^ T cells under siFoxp1 Agg‐exo co‐culture relatively low expressed CD44, CD25, and Ki67, compared to Agg‐exo, scale bar, 2 µm. L) Western blot results showed that CD4^+^ T cells induced by siFOXP1 Agg‐exo exhibited low levels of p‐STAT5. M,N) Immunofluorescence results showed a low level of expression of STAT5, Foxp1, and Foxp3 in the nucleus, meanwhile, revealing the low co‐localization of STAT5 and Foxp1, respectively, with Foxp3, scale bar, 2 µm. ns, not significant, ^*^p < 0.05; ^**^p < 0.01; ^***^p < 0.001; ^****^p < 0.0001.

To further explore the underlying mechanisms through which engineered Agg‐exos promote Treg differentiation, we performed proteomic analysis on the exosomal content of Agg‐exos. The proteomics analysis confirmed the expression of exosomal marker proteins (Figure S3A, Supporting Information), revealing 1152 proteins upregulated and 701 downregulated in Agg‐exo compared to Adh‐exo (Figure S3B, Supporting Information). GO analysis showed that upregulated proteins in Agg‐exo were highly enriched in nuclear‐associated cellular components, including the nucleus, nucleoplasm, and nucleolus, as well as biological processes such as RNA processing, mRNA splicing, and ribosomal biogenesis (Figure S3C,D, Supporting Information). These findings suggest that Agg‐exo preferentially carry nuclear‐associated proteins involved in gene regulation. While KEGG analysis further supported this by showing that Agg‐exo‐enriched proteins are significantly involved in pathways related to mRNA surveillance, RNA polymerase activity, and DNA replication, which are crucial for transcriptional regulation and immune cell function (Figure S3E, Supporting Information). Notably, FoxP1 is a key transcriptional regulator of Treg differentiation, playing an essential role in mRNA splicing, chromatin remodeling, and interaction with FoxP3.^[^ ^24^, ^43^ ^]^ Among the differentially expressed proteins, Foxp1 was significantly upregulated in Agg‐exos compared to Adh‐exos, directly linking it to the distinct functional properties of Agg‐exo, as confirmed by western blot analysis (**Figure** **5**A; Figure S4A, Supporting Information).

In CD4^+^ T cells treated with Agg‐exos, western blot results demonstrated a marked increase in phosphorylated STAT5 (p‐STAT5) expression (Figure 5B). Immunofluorescence analysis further revealed increased co‐localization and expression of STAT5 and Foxp3 in the nucleus (Figure 5C,D). Simultaneously, Foxp1 was highly expressed in the nucleus and showed significant co‐localization with Foxp3 (Figure 5E,F). These findings suggest that Foxp1 not only indirectly regulates Foxp3 expression by promoting STAT5 activation and nuclear translocation but also directly interacts with Foxp3 to enhance Treg differentiation.

To confirm the pivotal role of Foxp1, we silenced Foxp1 in MSCs using siRNA and generated siFoxp1 Agg‐exo (Figure 5G,H). CD4^+^ T cells treated with siFoxp1 Agg‐exo exhibited a significant reduction in Treg cell differentiation compared to those treated with Agg‐exo (Figure 5I), while no significant change in Th1, Th2, or Th17 cells (Figure S4B, Supporting Information). The expression of genes associated with Treg differentiation, as well as markers of Treg activity, was also significantly decreased in siFoxp1 Agg‐exo (Figure 5J,K). Furthermore, siFoxp1 Agg‐exo failed to enhance p‐STAT5 expression in CD4^+^ T cells (Figure 5L), and the co‐localization of STAT5 and Foxp3 in the nucleus was markedly reduced compared with Agg‐exo (Figure 5M; Figure S4C, Supporting Information). Likewise, Foxp1 expression and its nuclear co‐localization with Foxp3 were significantly diminished (Figure 5N; Figure S4C, Supporting Information). Additionally, the Treg suppression assay revealed that Tregs treated with siFoxP1 Agg‐exo exhibited a reduced suppressive capacity on Teff proliferation compared to those treated with Agg‐exo (Figure S4D, Supporting Information).

Given that abnormal B cell subset differentiation is closely linked to disease severity and closely interacts with dysregulated T cell differentiation, we conducted additional experiments to evaluate the effects of Agg‐exo and Foxp1 on B cell subpopulations associated with SLE progression. In vitro flow cytometry analysis of cultured B cells revealed that Agg‐exo reduced activated naïve B cells (aNBCs), double‐negative (DN) B cells, and plasma cells (PCs), while increasing non‐switched memory B cells (NSMs) compared to Adh‐exo (Figure S4E, Supporting Information). Notably, siFoxp1 Agg‐exo attenuated the inhibitory effect on PC differentiation, whereas other B cell subsets remained unaffected, suggesting that FoxP1 specifically contributes to PC suppression.

Collectively, these data suggest that Foxp1 is enriched in engineered Agg‐exos and plays a pivotal role in promoting Treg differentiation via the Foxp1/STAT5/Foxp3 axis.

### 2.6 Combined Engineering Strategy Generates Foxp1^high^ Agg‐Exos with Superior Therapeutic Efficacy in SLE

**Figure 6:**
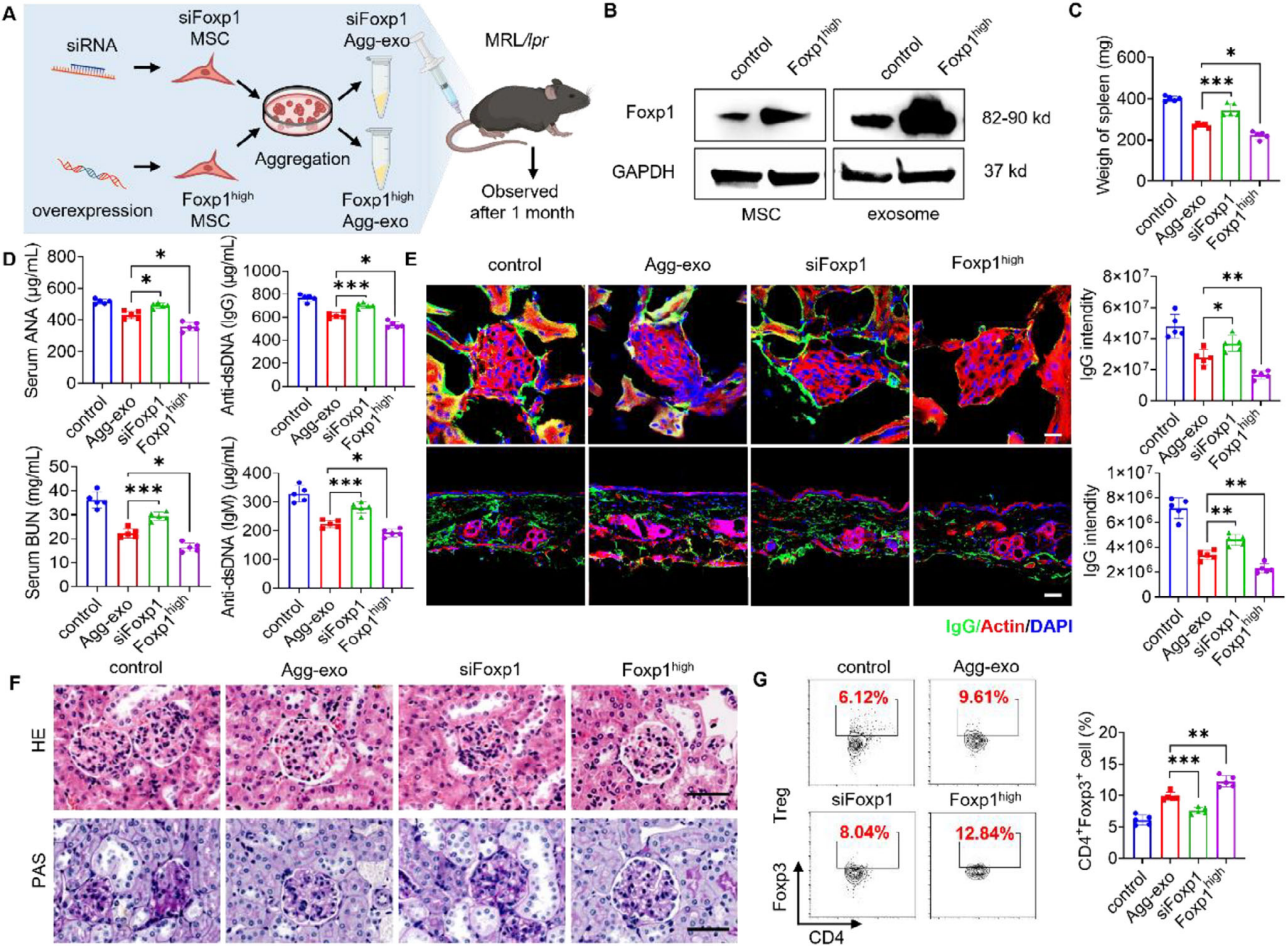
Dual‐engineered Foxp1^high^ Agg‐exos treatment of SLE. A) Schematic diagram showing our exosome treatment protocol for MRL/*lpr* mice and efficacy assessment. B) Western blot showed that the MSCs and derived exosomes significantly higher expressed Foxp1 after lentiviral transfection. C) In the spleen weight of MRL/*lpr*, siFoxp1 Agg‐exos reduced less weight of the spleen than the Agg‐exo group, while Foxp1^high^ showed an opposite effect, *n* = 5. D) The concentration of serum ANA, BUN, and dsDNA (IgG) in siFoxp1Agg‐exo declined less than the Agg group, compared to the control group, while Foxp1^high^ showed the opposite trend, *n* = 5. E) Confocal assay confirmed the IgG deposit degree in the kidney and skin of under exosome treatment, siFoxp1 Agg‐exos were significantly more than Agg‐exos, with an opposite trend observed in Foxp1^high^, *n* = 5; scale bar, 20 µm for kidney; scale bar, 50 µm for skin. F) H&E and PAS staining results showed siFoxp1 Agg‐exo significantly reduced the ability to weaken kidney damage, while Foxp1^high^ showed the opposite trend, scale bar, 100 µm. G) Flow cytometry showed that the ratio of CD4^+^Foxp3^+^ Treg cells was lower in the siFoxp1 Agg‐exo group and higher in Foxp1^high^, *n* = 5. ns, not significant, ^*^p < 0.05; ^**^p < 0.01; ^***^p < 0.001; ^****^p < 0.0001.

Our previous results have demonstrated that Foxp1 is a key component of engineered Agg‐exos in regulating Treg differentiation in vitro. To develop a therapeutic exosome with the inherent benefits of Agg‐exos and enhanced immunomodulatory capacity, we employed a dual‐engineering strategy combining gene editing and aggregation culture to construct Foxp1^high^ Agg‐exos (**Figure** **6**A,B). We also performed exosome characterization for siFoxp1 and Foxp1^high^ Agg‐exos, which retained the immune organ targeting ability and high yield of Agg‐exos (Figures S5 and S6A,B, Supporting Information). Foxp1^high^ Agg‐exos and siFoxp1 Agg‐exos were systemically administered to MRL/*lpr* mice separately for therapeutic efficacy observation (Figure 6A). The results indicated that the therapeutic effects of Foxp1^high^ Agg‐exos were significantly improved in MRL/*lpr* mice.

The spleen weight in the Foxp1^high^ Agg‐exo group was significantly reduced, compared to that of the Agg‐exo and siFoxp1 group (Figure 6C). ELISA results revealed markedly reduced levels of serum ANA, BUN, and dsDNA in the Foxp1^high^ Agg‐exo group, further supporting the enhanced therapeutic efficacy (Figure 6D). Furthermore, immunofluorescence results showed significantly diminished IgG deposition in the kidneys and skin of mice treated with Foxp1^high^ Agg‐exos compared to those treated with Agg‐exos and siFoxp1 Agg‐exos (Figure 6E). The renal pathology staining also demonstrated greater improvement in the Foxp1^high^ Agg‐exo group compared to Agg‐exo, while the siFoxp1 Agg‐exo group exhibited an opposite trend (Figure 6F).

Moreover, flow cytometry analysis revealed that Foxp1^high^ Agg‐exos had a stronger capacity on promoting Treg differentiation, as evidenced by a higher Treg ratio compared to Agg‐exos, while siFoxp1 exhibited the opposite trend, and the same tendency were observed on the CD69 ^+^ and CTLA‐4 ^+^ (Figure 6G; Figure S6C, Supporting Information). There were no notable changes in the proportions of Th17, Th1, or Th2 cells, consistent with the in vitro findings (Figure S6D, Supporting Information). Additionally, flow cytometry confirmed B cell subset changes consistent with in vitro findings, further supporting the therapeutic potential of Agg‐exo (Figure S6E, Supporting Information). However, siFoxp1 and FoxP1 ^high^ Agg‐exo differentially affected aNBCs and PCs compared to Agg‐exo, with PC modulation aligning with in vitro results (Figure S6E, Supporting Information). These results collectively suggest that the Foxp1^high^ Agg‐exos, constructed by the combined engineering strategy, offer superior therapeutic effects in ameliorating SLE symptoms in MRL/*lpr* mice. This efficacy is largely attributed to Foxp1‐mediated promotion of Treg differentiation and the innate advantages of Agg‐exos.

## 3 Discussion

The immunomodulatory effects of MSC‐exos have garnered significant attention in the treatment of autoimmune diseases. However, the limited yield of exosomes and the poor therapeutic specificity hinder their therapeutic efficacy and clinical application.^[^ ^44^ ^]^ In this study, we developed a novel dual‐engineering strategy combining aggregation culture and Foxp1 overexpression to overcome these limitations. Our engineered Foxp1^high^ Agg‐exos demonstrated significantly higher production than conventional culture, the unique immune‐targeting properties, and potent immunomodulatory capacity via the Foxp1/STAT5/Foxp3 axis, leading to substantial therapeutic improvements for SLE in MRL*/lpr* mice.

Exosome bioengineering for enhanced yield and therapeutic efficacy has been utilized to address the clinical application dilemma of traditional conventional MSC‐exos.^[^ ^45^ ^]^ One stable exosome engineering approach is manipulating cell culture conditions, such as cell aggregation‐based culture systems. The aggregation system can generate vesicles with high production and enriched bioactive cargo tailored for specific therapeutic applications.^[^ ^46^ ^]^ Several mechanisms have been proposed to explain the enhanced exosome production in aggregation culture: 1) Hypoxia in the spheroid core due to limited oxygen supply.^[^ ^47^ ^]^ 2) Reduced F‐actin expression leads to cytoskeletal tension and reorganization, altering matrix nanoscale mechanics and mechanical stress.^[^ ^48^ ^]^ 3) Increased autophagic flux in aggregated MSCs.^[^ ^49^ ^]^ 4) Transition from oxidative phosphorylation to glycolysis.^[^ ^50^ ^]^ Currently, engineering methods for inducing MSCs aggregation include the hanging drop method, forced‐aggregation, spontaneous assembly, chitosan films, and hyaluronic acid gel. Here, we utilized an ultra‐low attachment culture system to form MSC aggregates, yielding engineered Agg‐exos. This aggregation induction rapidly led to a 14‐fold increase in exosome production within 24–48 h, compared to traditional adherent culture. The robust exosome production of engineered Agg‐exo highlights aggregation culture as an efficient and fundamental platform for generating high‐quality engineered vesicles.

A remarkable finding in this study is the preferential biodistribution of engineered Agg‐exos in immune organs, such as the spleen, thymus, and lymph nodes. It was further supported by in vitro experiments that CD4^+^ T cells preferentially captured Agg‐exos. This unique immune‐targeting preference holds a critical advantage for the treatment of SLE, where immune dysregulation is central to pathogenesis. This finding is particularly significant in the current pursuit of more efficient and targeted therapies. The targeting capacity likely stems from the altered surface protein composition of Agg‐exo, which may be enriched with molecules conducive to immune cell interaction. Studies have reported that dendritic cell‐derived exosomes express high levels of ICAM‐1, which interacts with LFA‐1 on activated T cells to facilitate targeted uptake.^[^ ^12^ ^]^ Similarly, our proteomic sequencing analysis revealed a significant upregulation of ICAM‐1 in Agg‐exo, suggesting that the enhanced uptake of Agg‐exo by CD4⁺ T cells may be mediated through the ICAM‐1/LFA‐1 recognition pathway. Currently, efforts to achieve EV targeting include inserting coding sequences of ligands, core ligand fragments, or homing peptides between the signal peptide and the N‐terminus of a transmembrane protein maturation peptide.^[^ ^51^, ^52^, ^53^ ^]^ However, limitations such as instable expression and complex processes have hindered their widespread application. Notably, all kinds of exosomes tend to accumulate in the liver, highlighting the challenge of preventing off‐target delivery, which remains a significant obstacle in exosome‐based therapies.^[^ ^12^ ^]^ Our results demonstrated for the first time that Agg‐exos have immune‐targeting capability, offering a novel approach to achieving immune‐targeted delivery by aggregation engineering without complex surface engineering.

The depletion or dysfunction of Treg cells and the resulting breakdown of peripheral tolerance are recognized as critical pathogenic mechanisms in SLE.^[^ ^54^ ^]^ Prior studies have shown that MSC aggregates exhibit superior immunomodulatory effects, including enhancement of anti‐inflammatory mediators, suggesting the potential immunomodulatory ability of Agg‐exo.^[^ ^55^ ^]^ In our study, engineered Agg‐exos were preferentially captured by CD4^+^ T cells, further leading to increased Treg differentiation and function. Mechanistically, our proteomic analysis revealed that Foxp1 was significantly enriched in Agg‐exo. Foxp1 is ubiquitously expressed in normal tissues and has been implicated in B‐cell development, monocyte differentiation, and lung epithelial regeneration.^[^ ^23^, ^56^, ^57^ ^]^ Recent studies showed that Foxp1 prevents spontaneous T cell activation, preserves memory potential, and is critical for the development and differentiation of Treg cells.^[^ ^23^, ^26^ ^]^ Foxp1 plays a non‐redundant role in Treg cells by regulating Foxp3 chromatin binding and enhancing IL‐2 signaling, both of which are essential for maintaining Treg function.^[^ ^58^ ^]^ In our engineered Agg‐exo, Foxp1 promoted the phosphorylation and nuclear translocation of STAT5, and STAT5 co‐localized with Foxp3 in the nucleus to enhance its transcriptional activity. Additionally, Foxp1 itself translocated into the nucleus, where it directly co‐localized with Foxp3 to further promote its expression and support Treg differentiation. These effects were confirmed by siRNA‐mediated silencing of Foxp1 in exosomes. Here, we figure out that Foxp1 is the key factor in Agg‐exo‐mediated promotion of Treg differentiation, and propose an innovative Foxp1/STAT5/Foxp3 pathway underlying this process.

Our results indicate that Agg‐exo inhibits PCs differentiation via high FoxP1 expression, aligning with findings that FoxP1 represses PRDM1 (Blimp‐1), XBP1, and IRF4, key regulators of PC maturation.^[^ ^59^ ^]^ This suggests that Agg‐exo helps maintain a regulatory B cell phenotype by suppressing terminal differentiation. Additionally, FoxP1 is crucial for germinal center B cell regulation, and its downregulation is required for proper activation.^[^ ^60^ ^]^ The observed reduction in aNBCs, DN B cells, and PCs following Agg‐exo treatment suggests suppression of excessive B cell activation in SLE, further supported by the partial reversal seen with siFoxp1 Agg‐exo. The in vivo effects on aNBCs may result from Treg‐mediated suppression of B cell activation, as CD4⁺ T cells enhance pathogenic aNBCs activity in SLE.^[^ ^61^ ^]^ Given that Agg‐exo promotes Treg function, their impact on B cells likely occurs via FoxP1‐dependent immune modulation, highlighting their potential as an immunotherapeutic strategy in SLE.

Targeting Foxp1, the key protein with immunomodulatory functions in Agg‐exo, we employed a combined engineering approach. Foxp1 was overexpressed in MSCs via lentiviral transduction, followed by aggregation induction to produce Foxp1^high^ Agg‐exos. Our engineering exosomes capitalized on both the yield and immune‐targeting properties of Agg‐exo, together with the added advantage of Foxp1's potent immunoregulatory capabilities. Previous studies have utilized complex engineering approaches to achieve similar effects, such as overexpressing CD47 on MSCs and encapsulating miR‐21a to obtain CD47‐EVs in targeting therapy in cardiovascular diseases.^[^ ^62^ ^]^ Scaffold proteins can also be pre‐loaded into original cells to further access EV platforms for simple, editable therapeutic properties.^[^ ^17^ ^]^ However, similar engineering exosomes with such targeting capabilities still face the challenge of low yield. Our engineering strategy demonstrates a streamlined and effective approach to optimize MSC‐exos for autoimmune disease treatment, addressing both the yield and therapeutic specificity challenges that have hindered previous exosome‐based therapies.

In conclusion, our study reported a novel engineering approach to generate Foxp1^high^ Agg‐exo with highly improved yield, immune‐targeting biodistribution, and potent immunomodulatory effects via the Foxp1/STAT5/Foxp3 axis, which is unprecedented. Our engineered exosomes, with their immune‐targeting capability for Treg cells, demonstrated exceptional therapeutic efficacy in SLE and promising potential for other autoimmune diseases. Furthermore, the combined engineering strategy, based on aggregation‐induced exosome engineering, offers a streamlined and efficient platform pattern for developing engineered exosomes tailored to various diseases.

## 4 Experimental Section

### Animals and Ethics Statement

All animal experiments were conducted in accordance with protocols approved by the Institutional Animal Care and Use Committee of Sun Yat‐sen University (SYSU‐IACUC‐2024‐002028). B6.MRL‐Faslpr/J (MRL/*lpr*) mice were obtained from the Jackson Laboratory. Equal doses of different kinds of exosomes (1 × 10¹⁰ particles per mouse) were administered via tail vein injection. For the assessment of in vivo distribution, observations were made 24‐h post‐exosome treatment. To evaluate SLE symptoms, tissue collection was performed four weeks after exosome administration.

### Cell Culture

Human umbilical cord mesenchymal stem cells (UMSC) were isolated and cultured as described in previous studies. Briefly, UMSCs were obtained from full‐term cesarean sections with the informed consent of the donors. The cells were cultured in alpha minimum essential medium (α‐MEM, Invitrogen) supplemented with 20% fetal bovine serum (FBS, Gibco), 2 mM L‐glutamine (Invitrogen), 55 mm 2‐mercaptoethanol (Invitrogen), and 1% penicillin/streptomycin (Invitrogen). Depending on the experimental group, the cells were inoculated in either 10 cm culture flasks (Corning, Cambridge, MA, USA) or 10 cm ultra‐low‐attachment culture dishes (Corning) at 37 °C in a humidified atmosphere containing 5% CO_2_.

### Antibodies and Reagents

All antibodies, cytokines, kits, and other recourse used in this study were listed in Table S1 (Supporting Information).

### Isolation, Characterization and Labeling of Exosomes

The same amount of MSC was cultured in exosome‐depleted medium (complete medium depleted of FBS‐derived exosomes by overnight centrifugation at 100 000 g) for 48 h. Exosomes from culture supernatants were isolated by differential centrifugation, as described in the literature,^[^ ^63^ ^]^ at 300 g for 10 min, 2000 g for 10 min, 10000 g for 30 min and 100 000 g for 70 min, followed by washing with PBS and purification by centrifugation at 100 000 g for 70 min.

In each exosome preparation, characterization was conducted using transmission electron microscopy (TEM), nanoparticle tracking analysis (NTA), nano‐flow cytometry measurement (nFCM), and Western blot analysis to meet ISEV standards and ensure reproducibility.^[^ ^64^ ^]^ TEM (JEOL, Tokyo, Japan) was utilized to assess the morphology and ultrastructure of the exosomes. The Pierce BCA Protein Assay (Thermo Fisher Scientific) was employed to determine the protein concentration of the exosomes. NTA measured their quantity, size, and zeta potential using ZetaView PMX120 (Particle Metrix, Germany). Additionally, nFCM was used to evaluate the expression levels of exosomal markers CD63, CD9, and CD81. Specifically, exosomes were incubated with PE‐conjugated antibodies against CD63, CD9, and CD81 at 37 °C for 30 min, then detected using nFCM (NanoFCM, Xiamen, China). Purified exosomes were characterized by Western blot analysis using antibodies anti‐CD63, TSG101, Alix, and Calnexin.

DiR (Invitrogen, USA) and PKH‐26 (Sigma) was used for exosome labeling according to the manufacturer's instructions.

For in vivo therapeutic applications, freshly isolated exosomes were used immediately. For exosomes requiring storage, samples were maintained at −80 °C to preserve vesicle integrity and bioactivity.^[^ ^65^ ^]^ Stored exosomes were resuspended in PBS. Before use, frozen exosomes were thawed on ice to prevent structural degradation and maintain functional stability, following best practices for exosome recovery.^[^ ^66^ ^]^

### Western Blotting

Total protein was extracted using a protein extraction kit (Thermo, Rockford, IL, USA) according to the manufacturer's instructions. A total of 20 µg of protein from each sample was loaded onto an SDS‐PAGE gel and subsequently transferred to a PVDF membrane (Millipore). The membrane was then blocked for 1 h before being incubated overnight at 4 °C with primary antibodies. Following this, the membrane was incubated with HRP‐conjugated secondary antibodies at room temperature for 1 h. After incubation, the membrane was subsequently rinsed with TBST for 10 min × 3 times. Finally, immunoreactive proteins were visualized using High Sensitivity ECL Substrate Kit (Abcam) and detected using a gel imaging system.

### In Vivo Imaging System (IVIS) Analysis

The in vivo distribution of exosomes was analyzed using IVIS imaging. Following administration of DiR‐labeled exosomes, mice were sacrificed after 24 h, and their organs were collected for IVIS analysis. Fluorescence intensity was measured to assess exosome accumulation levels in various organs.

### Immunofluorescence Staining and Histology

Frozen sections and cell samples were fixed at room temperature in 4% paraformaldehyde (Sigma–Aldrich) for 20 min. Subsequently, samples were stained with primary antibodies followed by secondary antibodies. Finally, samples were sealed with DAPI mounting medium and placed on adhesive microscope slides for imaging. Frozen kidney and skin tissue sections were prepared and stained with Alexa Fluor 488–conjugated anti‐mouse IgG; the fluorescence intensity was then used to evaluate IgG deposition.

For histological evaluation, renal tissues from different groups were fixed overnight in 4% paraformaldehyde (Sigma–Aldrich). Paraffin sections were then prepared and stained with hematoxylin and eosin (H&E) and periodic acid‐Schiff (PAS) to assess renal damage.

### Flow Cytometry

For intracellular cytokine staining, cells were stimulated for 4 h with phorbol 12‐myristate 13‐acetate (PMA, 50 ng mL^−1^), ionomycin (1 µg mL^−1^), and monensin (GolgiStop; 1 µg mL^−1^). After surface marker staining, the cells were permeabilized using a FoxP3/transcription factor staining kit (Thermo Fisher, CA, USA) and subsequently stained intracellularly with the following antibodies: APC anti‐mouse CD4, FITC anti‐mouse IFN‐γ, PE anti‐mouse IL‐4, PE anti‐mouse IL‐17, and PE anti‐mouse Foxp3. To assess the apoptotic rate, cells were stained with FITC anti‐Annexin V and PE‐Cy7 anti‐7AAD for 15 min at room temperature. Flow cytometry analysis was then conducted using a NovoCyte instrument (Agilent Technologies, CA, USA). The gating strategy for the identification of immune cell subpopulations was shown in Supplementary Figure S7 (Supporting Information).

### Treg Cell Differentiation

Naïve CD4^+^ T cells were isolated from the spleen by positive selection using the mouse naïve CD4^+^ T cell isolation Kit (BD) according to the manufacturer's protocol. Cells were activated with plate‐bound anti‐mouse CD3 (2 µg mL^−1^) and anti‐mouse CD28 (2 µg mL^−1^) in RPMI 1640 medium supplemented with 10% FBS, 1 mM L‐glutamine and 50 µM 2‐mercaptoethanol, 100 U mL^−1^ penicillin, and 100 µg mL^−1^ streptomycin. For Treg cell differentiation, TGF‐β1 (10 ng mL^−1^; R&D Systems, USA) and IL‐2 (100 U mL^−1^; Peprotech, USA) to induce Treg cell conversion were added to cell cultures.^[^ ^63^ ^]^ Cells were supplemented with fresh media on day 3 and analyzed on day 5.

### siRNA Transfection

For siRNA transfection, Foxp1 siRNA (Ribo, China) was utilized to transfect MSCs using Lipofectamine RNAiMAX transfection reagent (Thermo Fisher, USA) according to the manufacturer's instructions. Non‐targeting control siRNA (Ribo, China) served as a negative control. Transfection efficiency was assessed via Western blotting.

### RNA Extraction, Reverse Transcription, and RT‐qPCR

RNA was extracted from cells using NucleoZOL reagent (Gene Company Limited) and subsequently reverse‐transcribed into complementary DNA (cDNA) with PrimeScript RT Master Mix (TaKaRa, Ltd., Osaka, Japan). Gene expression levels were quantified via real‐time polymerase chain reaction (RT‐qPCR) conducted on a Bio‐Rad CFX96 detection system (Roche, Sweden) utilizing Hieff qPCR SYBR Green Master Mix (Yeasen, Shanghai, China). The oligonucleotides for qPCR analysis were listed in Table S2 (Supporting Information).

### LC‐MS/MS Analysis

Mass spectrometry was conducted using an Orbitrap Q Exactive HF‐X mass spectrometer integrated with an UltiMate 3000 LC system (Thermo Fisher). The samples were reconstituted in formic acid and loaded into a precolumn, which was then connected to a microcapillary analytical column. High‐energy collisional dissociation (HCD) tandem mass spectrometry (MS/MS) spectra were acquired in data‐dependent mode utilizing the top‐20 method. The resulting raw data files were analyzed against the Homo sapiens UniProt canonical database using pFind 3.0 studio (http://pfind.ict.ac.cn/software/pFind3/index.html).

### Plasmid Transfection

The plasmids targeting Foxp1 were purchased from FulenGen (Guangzhou, China). All plasmids were transfected with lipofectamine 3000 (Invitrogen, Carlsbad, CA, USA) according to the manufacturer's instructions.

### Statistical Analysis

Data normality was assessed using the Shapiro‐Wilk test. Outliers were identified using Grubbs’ test and removed if statistically justified. Data were normalized as necessary before analysis. Comparisons between two groups were performed using independent unpaired two‐tailed Student's t‐tests, while one‐way ANOVA with Tukey's post‐hoc test was used for multiple‐group comparisons. A P‐value < 0.05 was considered statistically significant. All experiments were conducted in biological triplicate (n = 3 or more) unless otherwise specified, and results were presented as mean ± standard deviation (SD). Statistical analyses were conducted using GraphPad Prism 7 (GraphPad Software, USA).

## Conflict of Interest

The authors declare no conflict of interest.

## Author Contributions

L.N. and Q.O. contributed equally to this work. L.N. and Q.O. contributed to the conception of the project, study design, execution of experimental procedures, and drafting of the manuscript. Q.R. and Z.L. were involved in data acquisition, analysis, and interpretation. H.C. and F.L. contributed to data interpretation. X.M. provided essential resources. S.S. was responsible for project conception, experimental design, manuscript preparation, resource provision, and overall supervision. Z.C. and W.T. contributed to manuscript writing and supervision. All authors reviewed and approved the final version of the manuscript.

## Supporting information



Supporting Information

Supporting Information

## Data Availability

Research data are not shared.
