# Supplementary material for: Engineered Foxp1high Exosomes Ameliorates Systemic Lupus Erythematosus
Source: Adv Sci (Weinh). 2025 Jul 3;12(37):e15712. doi: 10.1002/advs.202415712 (PMC12499428; doi:10.1002/advs.202415712)
Supplement: Supplementary file 1 — Supporting Information [file ADVS-12-e15712-s002.docx]

**Supporting Information**

**Engineered Foxp1^high^ Exosomes Ameliorates Systemic Lupus Erythematosus**

Luhan Niu^1,#^, Qianmin Ou^1,#^, Qianhui Ren^1^, Zhengshi Li^1^, Hongcheng Chen^1^, Fangcao Lei^1^, Xueli Mao^1^, Songtao Shi^1,2^, Zetao Chen^1,*^, Teng Wei^1,*^

^1^South China Center of Craniofacial Stem Cell Research, Hospital of Stomatology, Sun Yat-sen University, Guangdong Provincial Key Laboratory of Stomatology, Guangzhou 510080, China

^2^Center for Stem Cell Biology and Tissue Engineering, Key Laboratory for Stem Cells and Tissue Engineering, Ministry of Education, Sun Yat-Sen University, Guangzhou, China

***CORRESPONDING AUTHORS**:

Zetao Chen, Hospital of Stomatology, Sun Yat-sen University and Guangdong Provincial Key, Laboratory of Stomatology, Guangzhou 510055, China. Tel: +86-20-83752769. Email: [chenzet3@mail.sysu.edu.cn](mailto:chenzet3@mail.sysu.edu.cn).

Wei Teng, Hospital of Stomatology, Sun Yat-sen University and Guangdong Provincial Key, Laboratory of Stomatology, Guangzhou 510055, China. Tel: +86-18988882233. Email: tengwei@mail.sysu.edu.cn


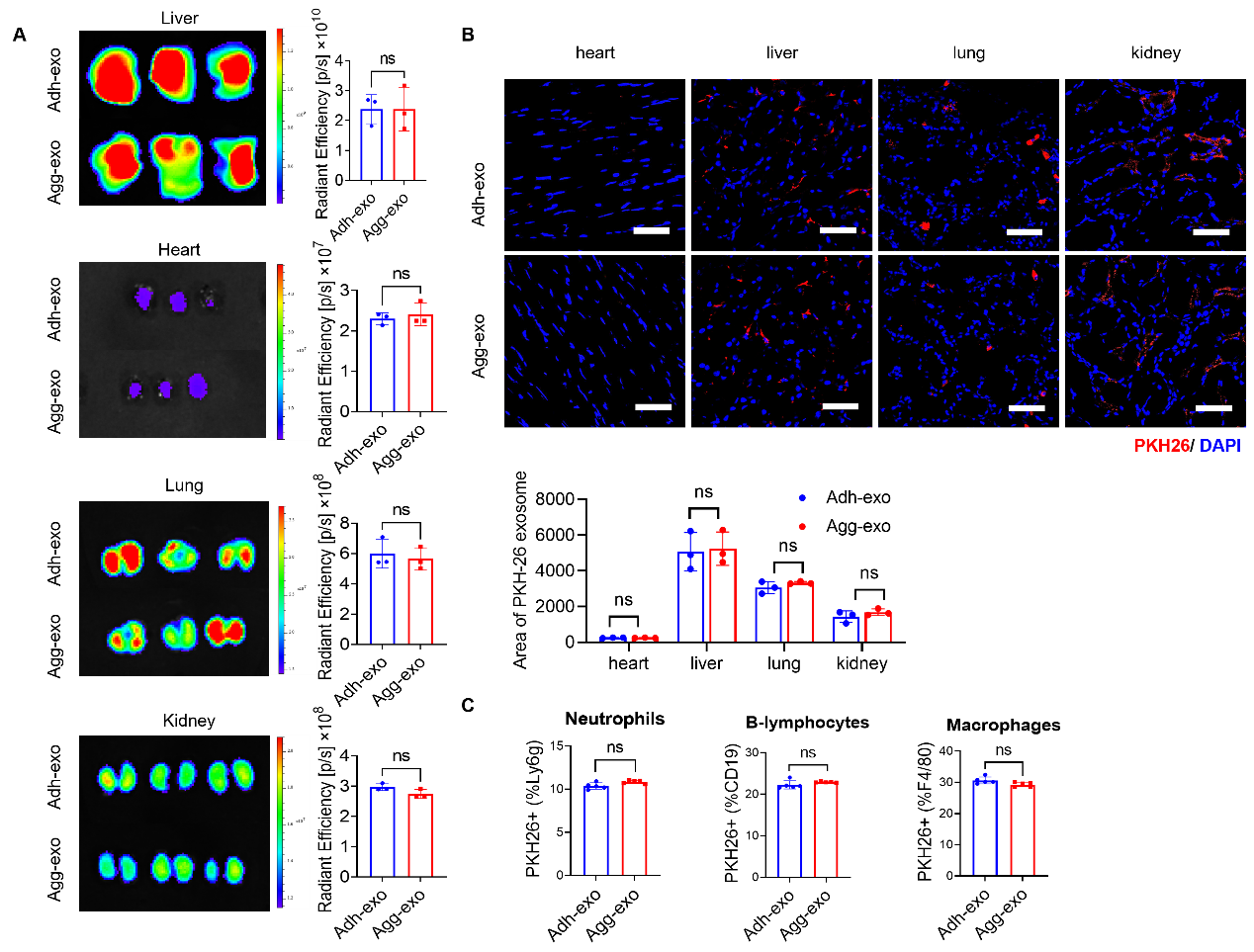


**Figure S1. Organ distribution of Agg-exos in MRL*/lpr* mice. (A and B)** IVIS analysis **(A)** and immunofluorescence staining of frozen tissue sections **(B)** showed that when DiR/PKH26-labelled exosome was systemically infused into MRL/*lpr* mice for 24 h, there was no significant difference between Agg-exos and Adh-exos in the distribution of liver, lung, heart and kidney, n = 3, scale bar, 50 μm. **(C)** Flow cytometry analysis shows that neutrophils, B lymphocytes, and macrophages show no significant differences in exosome uptaken in vivo, n = 5. ns, not significant.


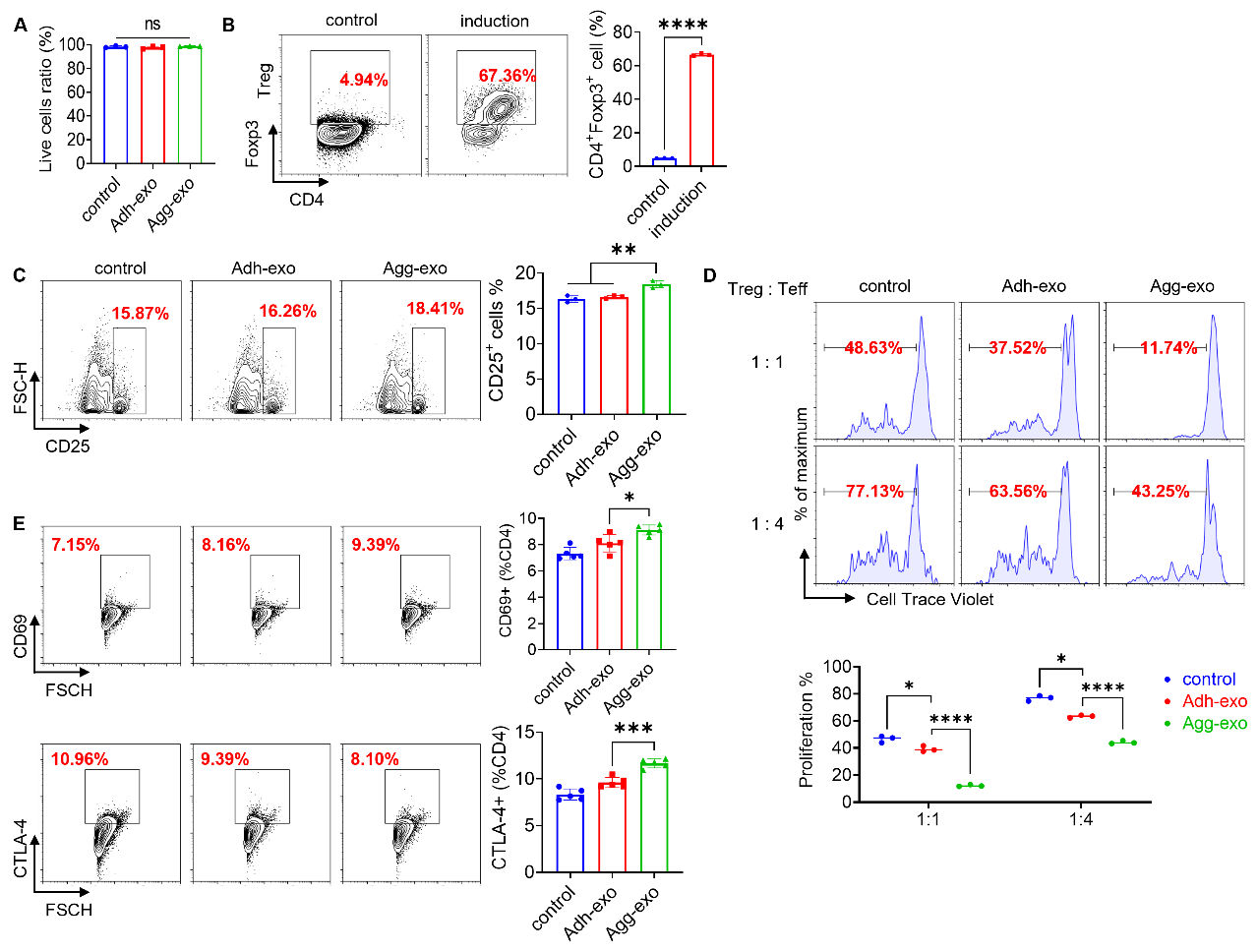


**Figure S2. CD4^+^ T cell culture in vitro and Treg induction**. **(A)** Flow cytometry showed that the ratio of cells with A5^-^7aad^-^ CD4^+^ T cells were more than 95% during Treg induction in vitro, n = 3. **(B)** After Treg differentiation induction in vitro, Flow cytometry resulted that the ratio of Foxp3^+^CD4^+^ Treg cells significantly higher, n = 3. **(C)** Flow cytometry of CD4^+^ T cells showed that CD4^+^ T cells under Agg-exo co-culture highly expressed CD25, n = 3. **(D)** In vitro Treg suppression assay: Tregs were co-cultured with CFSE-labeled PBMCs at different ratios, and proliferation was assessed after 72 hours. Both Agg-exo and Adh-exo inhibited PBMC proliferation compared to the control group, with Agg-exo exhibiting significantly stronger suppression than Adh-exo, n=3. **(E)** Flow cytometry analysis shows that one month after systemic exosome infusion in MRL/*lpr* mice, the ratio of CD69^+^ and CTLA-4^+^ T cells in the spleen was significantly higher in the Agg-exo group compared to the Adh-exo, n = 5. ns, not significant, **P*<0.05; ***P*<0.01; ****P*<0.001, *****P*<0.0001.


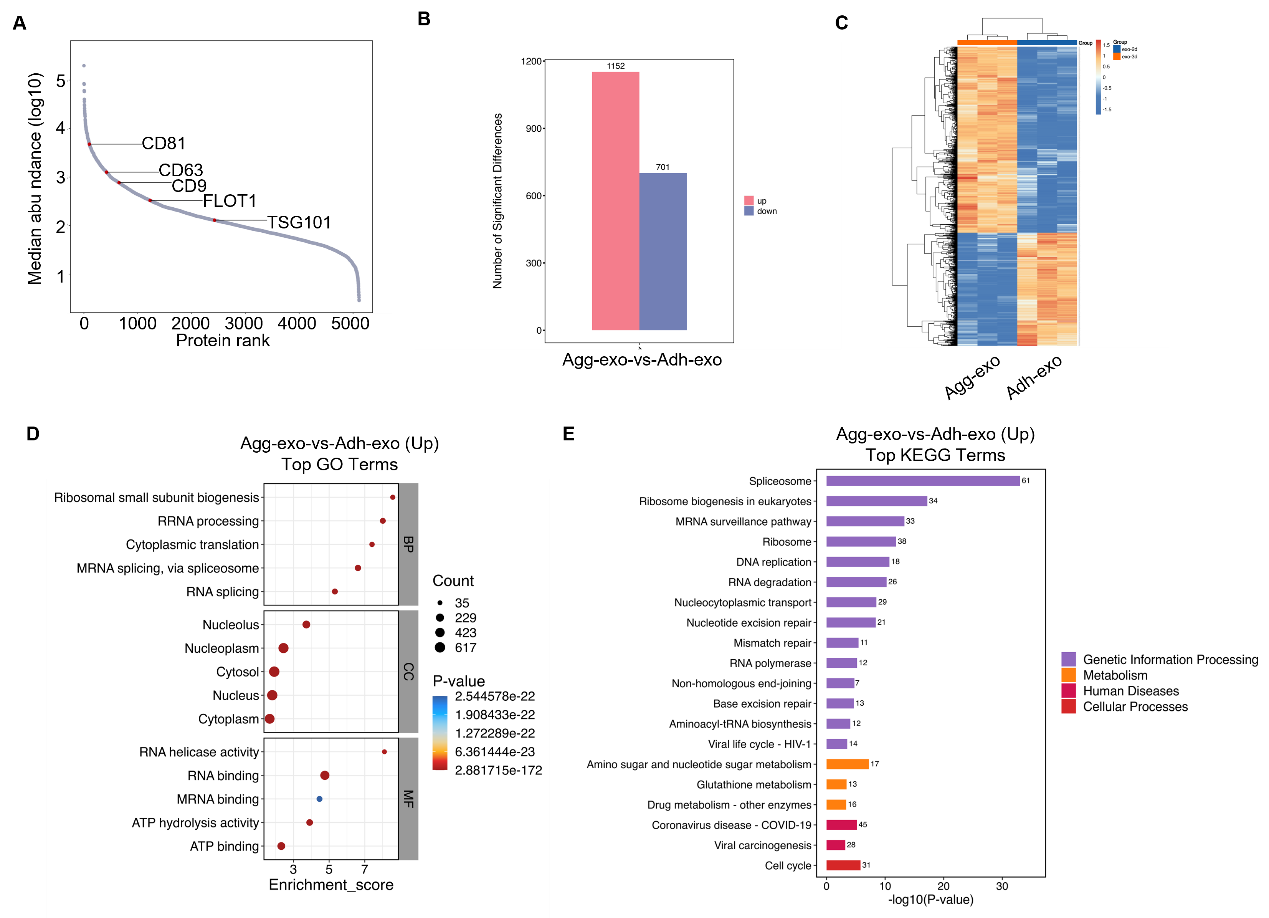


**Figure S3. High-throughput proteomic analysis of Agg-exos.** **(A)** The samples of Agg-exos and Adh-exos both Protein high abundance expression of exosomal marker profiles for each**. (B)** A bar chart illustrates the significantly upregulated and downregulated proteins in Agg-exos compared to Adh-exos. **(C)** Hierarchical clustering heatmap represent the hierarchical clustering analysis of Agg-exos and Adh-exos. Rows represent individual genes, and columns represent biological replicates, indicating differential molecular signatures based on culture condition, n = 3. **(D)** GO enrichment analysis of molecular function of proteins upregulated in Agg-exos compared to Adh-exos. **(E)** KEGG pathway analysis of indicated the upregulated differential proteins of Agg-exos annotated to the pathway group.


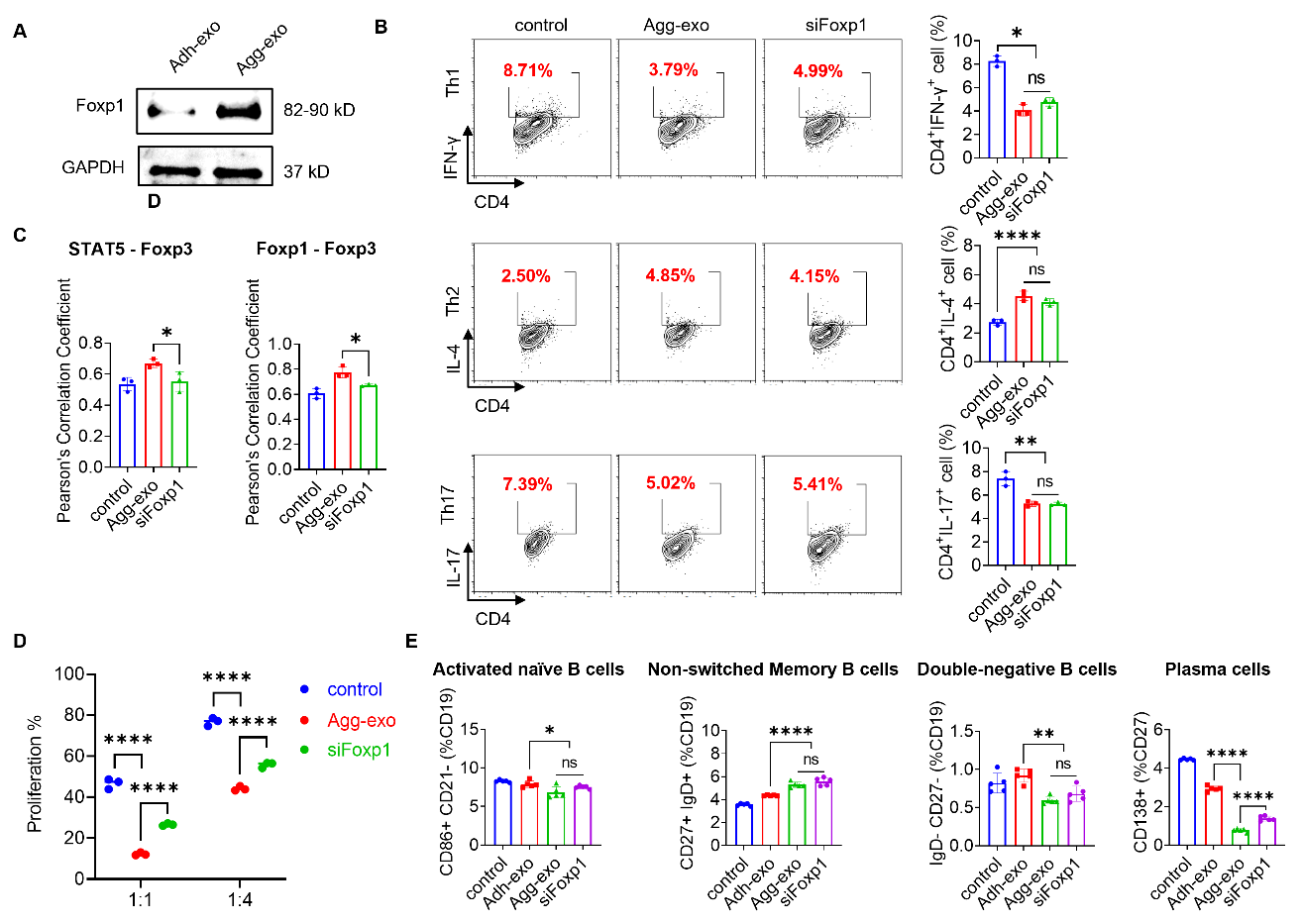


**Figure S4. Foxp1 enriched in Agg-exos and regulate the Treg differentiation. (A)** Western blot showed that Foxp1 highly expressed in Agg-exos compared to Adh-exos. **(B)** Flow cytometry of CD4^+^ T cells showed no significant difference on the ratio of Th1, Th2, and Th17 cells between Agg-exos and siFoxp1 Agg-exos treatment. **(C)** Statistical analysis of the co-localization of Foxp3 with STAT5**,** and Foxp1 respectively, in the nucleus showed a significant decrease in co-localization in the siFoxp1 group, n = 3. **(D)** In vitro Treg suppression assay, siFoxp1 Agg-exo exhibiting significantly stronger suppression than Agg-exo, n = 3. **(E)** Flow cytometry analysis shows that 72 hours after co-culture with exosomes, Agg-exo significantly reduced aNBCs, DN B cells, and PCs while increasing NSMs compared to Adh-exo. And siFoxp1 Agg-exo weakened the inhibitory effect on PC differentiation, while other subsets remained unchanged, n = 3. ns, not significant, **P*<0.05; ***P*<0.01; ****P*<0.001, *****P*<0.0001.


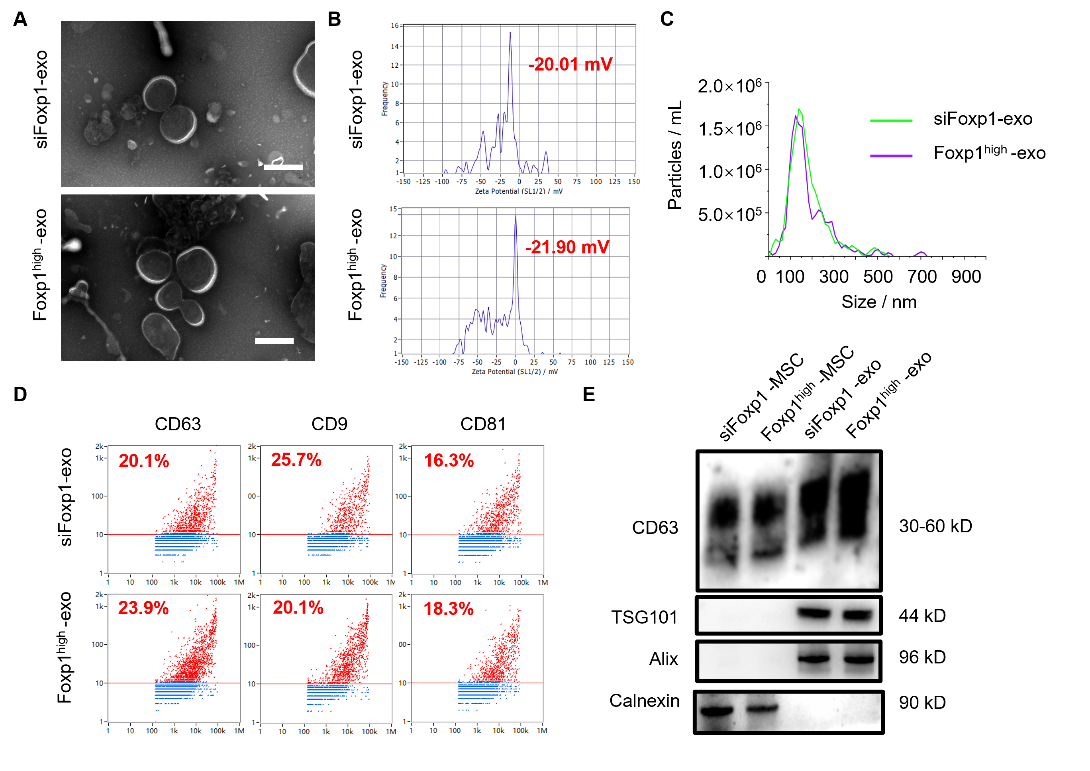


**Figure S5.** **Characterization of siFoxp1 and Foxp1 high Agg-exo.** **(A)** TEM showed that the isolated siFoxp1 and Foxp1 high Agg-exo presented an oval shape and a typical cup-shaped structure, scale bar, 200 nm. **(B and C)** NTA particle size analysis showed that the isolated siFoxp1 and Foxp1 high Agg-exo particle sizes were concentrated at <150 nm, and the average potentials of both were -38.07 mV and -42.57 mV, respectively. **(D)** NanoFCM results showed that both siFoxp1 and Foxp1 high Agg-exo highly expressed exosome surface markers: CD63, CD9 and CD81. **(E)** The western blot results showed that both siFoxp1 and Foxp1 high Agg-exo expressed high levels of CD63, TSG101 and Alix, and low levels of calnexin.


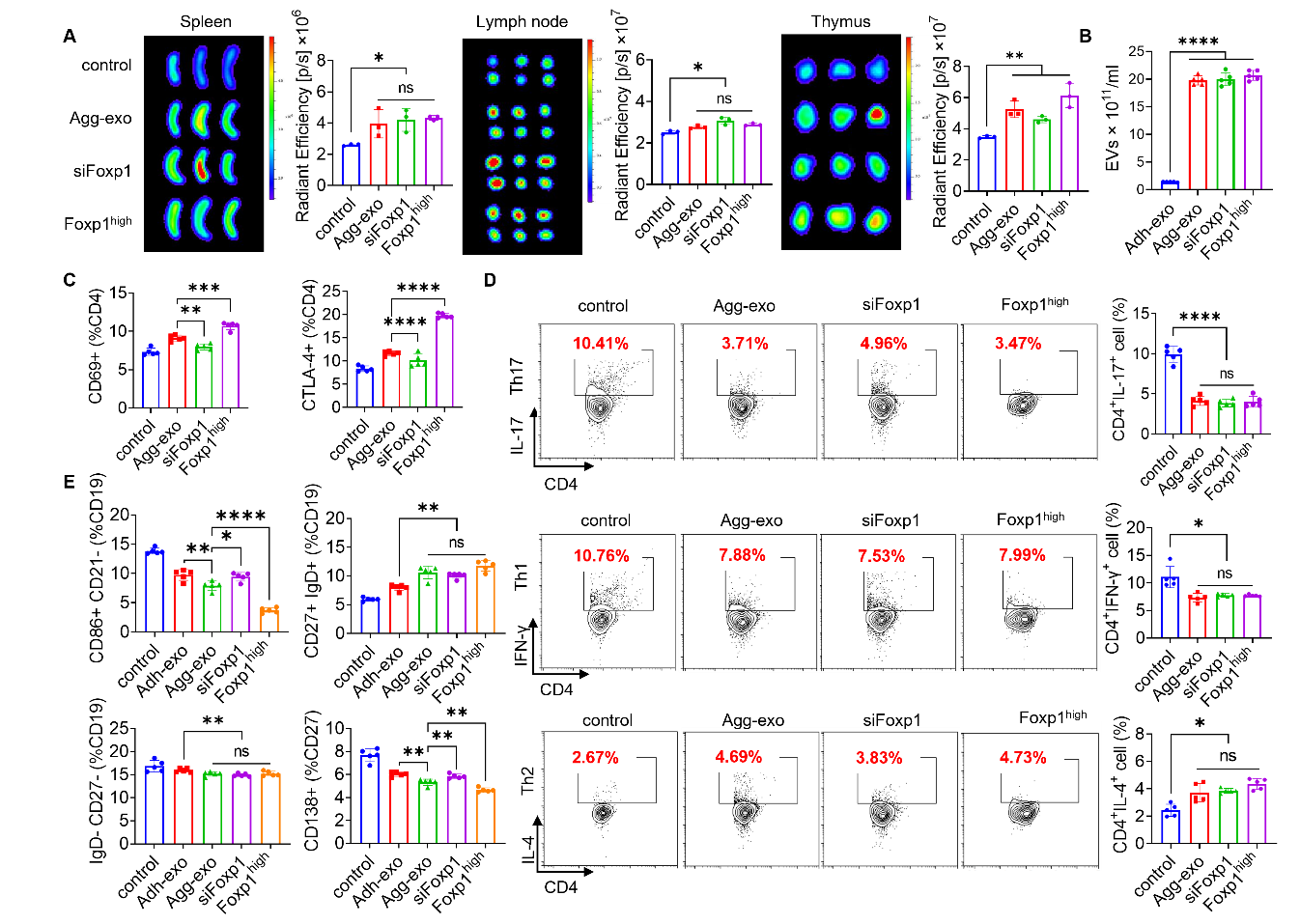


**Figure S6. Foxp1^high^ Agg-exos’ biodistribution and influence on T cell differentiation in MRL/*lpr* mice.** **(A)** IVIS analysis showed that, when DiR-exosomes were systemically infused into MRL/*lpr* mice for 24 h, the deposition of siFoxp1 and Foxp1^high^ Agg-exo in the spleen, lymph node, and thymus was higher than that of control, the same with Agg-exo, n = 3, scale bar, 50 μm. (**B**) The nanoparticle tracking analysis (NTA) results showed that the production of siFoxp1 and Foxp1^high^ Agg-exo were much higher than Adh-exo, n = 5. **(C)** Flow cytometry analysis shows that one month after systemic exosome infusion in MRL/lpr mice, the ratio of CD69^+^ and CTLA-4^+^ T cells in the spleen were significantly reduced in the siFoxp1 group, whereas were notably increased in the Foxp1 ^high^ group, n = 5 **(D)** Flow cytometry of spleen T cell showed no significant difference on the ratio of Th17, Th1, Th2 cells among Agg-exos, siFoxp1 and Foxp1^high^ treatment in MRL*/lpr* mice, n = 5. **(E)** Flow cytometry of in vivo B cells after one month of exosome treatment revealed that Agg-exo limited the expansion of aNBCs, DN B cells, and PCs while increasing NSMs compared to Adh-exo. Additionally, siFoxp1 Agg-exo increased aNBCs and PCs, while Foxp1 ^high^ Agg-exo reduced them compared to Agg-exo. No significant differences were observed among Agg-exo, siFoxp1, and Foxp1 ^high^ groups in NSMs and DN B cells, n=5. ns, not significant, **P*<0.05; ***P*<0.01; ****P*<0.001, *****P*<0.0001.


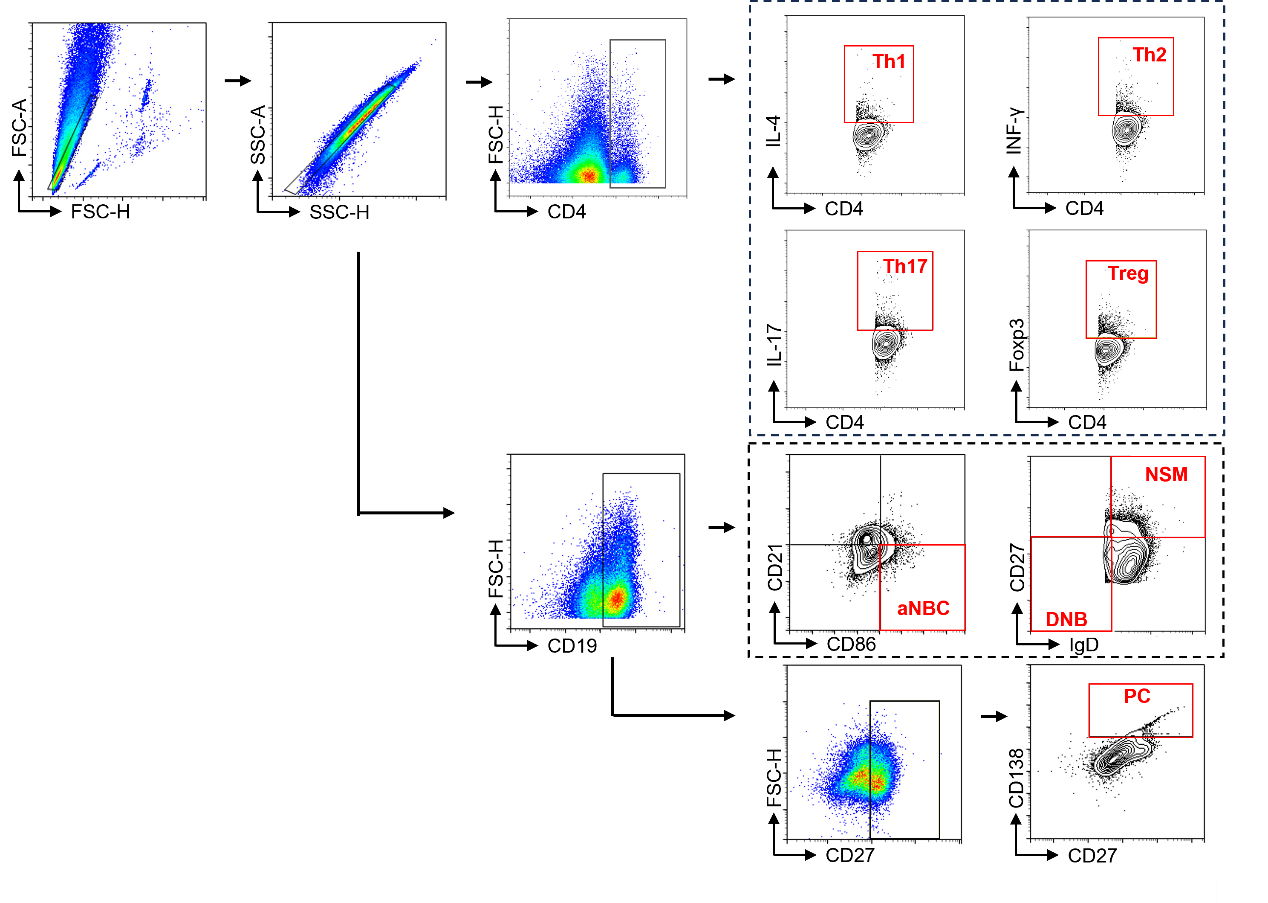
**Figure S7 Flow cytometry gating strategy for immune cell subpopulations.** Cells were first gated based on FSC-A vs SSC-A to exclude debris and identify intact cells according to their size and granularity. CD4⁺ T cells were gated and further subdivided into Th1 (CD4⁺IL4⁺), Th2 (CD4⁺INF-γ⁺), Th17 (CD4⁺IL-17⁺), and Treg (CD4⁺Foxp3⁺) cells. B cell subsets were gated from CD19⁺ populations, and defined as follows: aNBC (CD86^+^CD21⁺), NSM (CD27^+^IgD^+^), DNB (CD27^-^IgD^-^), PC (CD27^+^CD138^+^).

**Table S1. Reagents and resources table.**

| **Reagent or resource** | **Source** | **Identifier** |  |
| --- | --- | --- | --- |
| **Antibodies** |  |  |  |
| FITC anti-human CD63 | Biolegend | 353005 |  |
| PE anti-human CD63 | Biolegend | 353003 |  |
| PE anti-human CD9 | Biolegend | 312105 |  |
| PE anti-human CD81 | Biolegend | 349505 |  |
| PE Anti-Mouse CD3 | Biolegend | 100408 |  |
| APC anti-mouse CD4 | Biolegend | 100408 |  |
| PE anti-mouse IL-4 | Biolegend | 504104 |  |
| FITC anti-mouse IFN-γ | Biolegend | 505806 |  |
| PE anti-mouse IL-17 | Biolegend | 5069-4 |  |
| FITC anti-mouse CD25 | Biolegend | 102006 |  |
| PE anti-mouse Foxp3 | Biolegend | 320008 |  |
| PE anti-mouse CD44 | Biolegend | 553134 |  |
| PE anti-mouse/rat/human FOXP3 | Biolegend | 320008 |  |
| APC anti-mouse CD19 | Biolegend | 152409 |  |
| FITC anti-mouse CD27 | Biolegend | 124209 |  |
| Pacific Blue^TM^ anti-mouse IgD | Biolegend | 405711 |  |
| PE anti-mouse CD138 | Biolegend | 112503 |  |
| PE anti-mouse CD86 | Biolegend | 159203 |  |
| FITC anti-mouse CD21 | Biolegend | 123407 |  |
| FITC Annexin V | Biolegend | 640906 |  |
| Alexa Fluor® 647 Mouse anti-Ki-67 | BD | 558615 |  |
| Anti-GAPDH | CST | 1974S |  |
| Anti-CD63 | CST | 5810S |  |
| Anti-TSG101 | CST | 1739S |  |
| Anti-Alix | Abcam | ab275377 |  |
| Anti-Calnexin | Santa | sc-23954 |  |
| Anti-STAT5 | Zenbio | 381427 |  |
| Anti-p-STST5 | Zenbio | 381125 |  |
| Anti-Foxp3 | Zenbio | 200501-7H9 |  |
| Anti-Foxp1 | CST | 4402T |  |
| LEAF™ Purified anti-mouse CD3ε | Biolegend | 100302 |  |
| LEAF™ Purified anti-mouse CD28 | Biolegend | 102116 |  |
| Alexa Fluor™ 568 goat anti-rabbit IgG(H+L) | Invitrogen | A11036 |  |
| Alexa Fluor™ 488 goat anti-rabbit IgG(H+L) | Invitrogen | A11008 |  |
| Alexa Fluor™ 568 goat anti-mouse IgG(H+L) | Invitrogen | A11004 |  |
| Alexa Fluor™ 488 goat anti-mouse IgG(H+L) | Invitrogen | A11001 |  |
| Mouse-IgGκ BP-HRP | Santa Cruz Biotechnology | SC-516102 |  |
| Goat anti-Rat IgG (H+L) Secondary Antibody, HRP | Thermo Fisher Scientific. | 31470 |  |
| Mouse-IgGκ BP-HRP | Santa Cruz Biotechnology | sc-516142 |  |
| Mouse anti-rabbit IgG-B | Santa Cruz Biotechnology | sc-2491 |  |
| Mouse anti-rabbit IgG-HRP | Santa Cruz Biotechnology | sc-2357 |  |
| **Cytokines and other kits** | |  |  |
| Recombinant Murine IL-2 | | PeproTech | P04351 |
| Recombinant Mouse TGF-β Protein | | R&D | 7666-MB-005 |
| Alexa Fluor™ 555 phalloidin | | Thermo Fisher Scientific | A34055 |
| CellMask™ Deep Red Actin Tracking Stain | | Thermo Fisher Scientific | A57245 |
| 7-AAD | | BD Pharmingen | 559925 |
| 10X Annexin V Binding Buffer | | BD Pharmingen | 556454 |
| PKH26 Red Fluorescent Cell Linker kit | | Sigma-Aldrich | PKH26GL-1KT |
| Mounting Medium with DAPI | | Abcam | ab104139 |
| RIPA Lysis Buffer System | | Santa Cruz | sc-24948 |
| Dexamethasone | | Sigma-Aldrich | D4902 |
| Staurosporine | | Enzo Life Sciences | ALX-380-014 |
| TritonTM X-100 | | Sigma-Aldrich | X100-100ML |
| α-MEM Medium | | Invitrogen | 12571-048 |
| RPMI 1640 Medium | | biosharp | BL303A |
| Fetal bovine serum (FBS) | | Gibco | 10270-106 |
| L -glutamine | | Invitrogen | 35050-061 |
| 2-mercaptoethanol | | Invitrogen | 21985-023 |
| Penicillin-Streptomycin | | Invitrogen | 15140-122 |
| TrypLETM Express Enzyme | | Invitrogen | 12605-010 |
| Lipofectamine™ RNAiMAX Transfection Reagent | | Invitrogen | 13778 |
| Opti-MEM Medium | | Invitrogen | 31985070 |

**Table S2. Primers used in this study.**

| **Gene** | **Primer sequence (5’-3’)** |
| --- | --- |
| Foxp1-F | ATGATGCAAGAATCTGGGACTG |
| Foxp1-R | AGCTGGTTGTTTGTCATTCCTC |
| Foxp3-F | CACCTATGCCACCCTTATCCG |
| Foxp3-R | CATGCGAGTAAACCAATGGTAGA |
| Il2ra-F | CACTACGAGTGTATTCCGGGA |
| Il2ra-R | TCGGTGGTGTTCTCTTTCATCT |
| Tgfb1-F | CCACCTGCAAGACCATCGAC |
| Tgfb1-R | CTGGCGAGCCTTAGTTTGGAC |
| Ctla4-F | TTTTGTAGCCCTGCTCACTCT |
| Ctla4-R | CTGAAGGTTGGGTCACCTGTA |
| GAPDH-F | TCAGCAATGCCTCCTGCAC |
| GAPDH-R | TCTGGGTGGCAGTGATGGC |
