# Supplementary material for: Engineered Foxp1high Exosomes Ameliorates Systemic Lupus Erythematosus
Source: Adv Sci (Weinh). 2025 Jul 3;12(37):e15712. doi: 10.1002/advs.202415712 (PMC12499428; doi:10.1002/advs.202415712)

Unprocessed Western Blot Images for Manuscript advs.202415712R2

Figure 1J

CD63

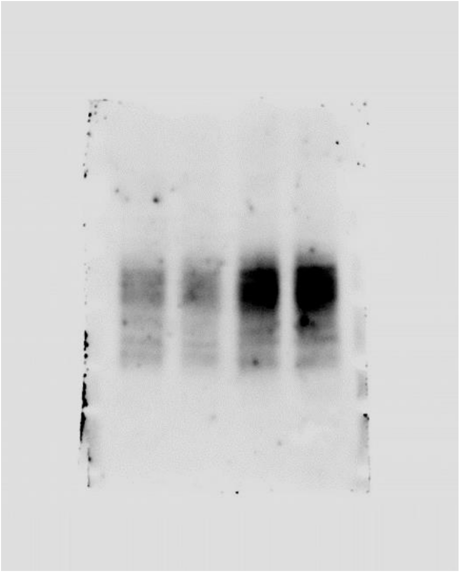

TSG101

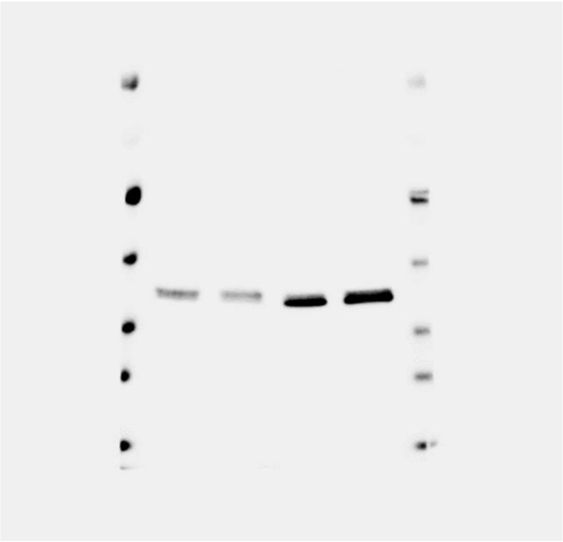

Alix

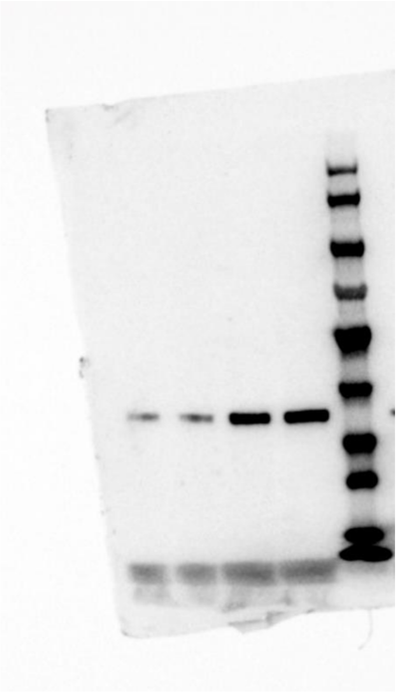

Calnexin

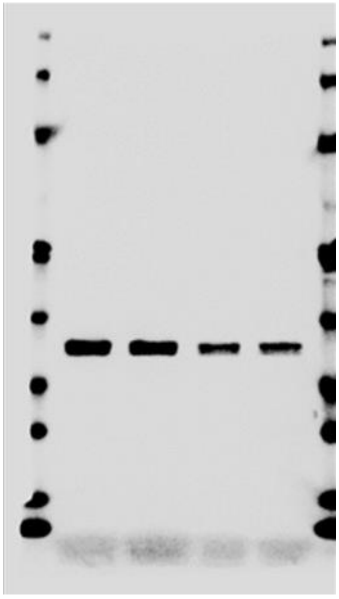

Figure 5B

STAT5

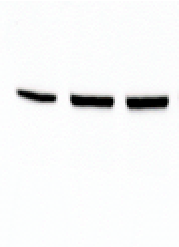

P-STAT5

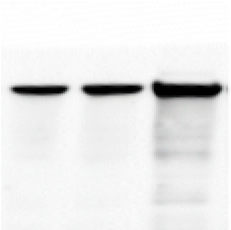

GAPDH

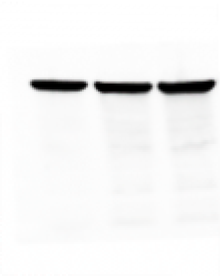

Figure 5H

MSC-Foxp1

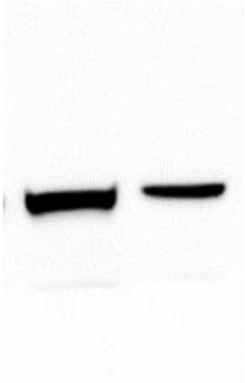

exosome-Foxp1

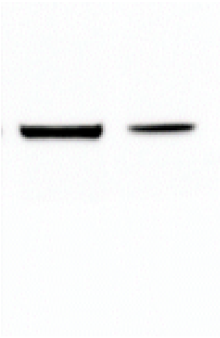

MSC-GAPDH

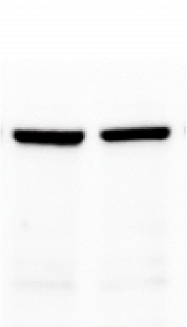

exosome-GAPDH

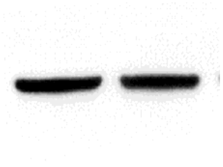

Figure 5L

STAT5

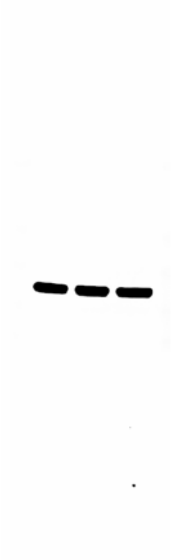

P-STAT5

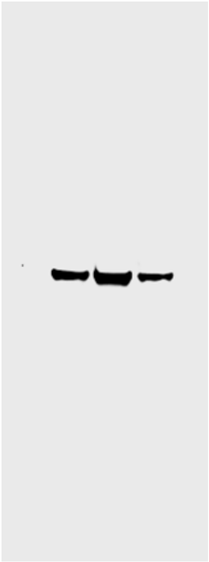

GAPDH

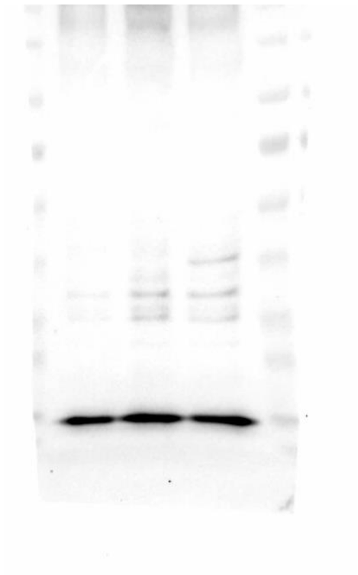

Figure 6B

MSC-Foxp1

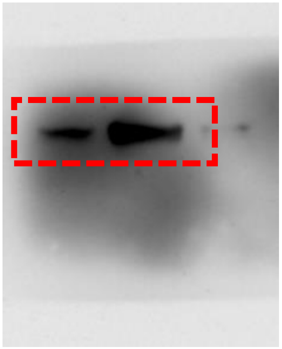

exosome-Foxp1

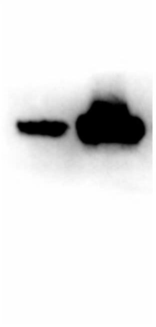

MSC-GAPDH

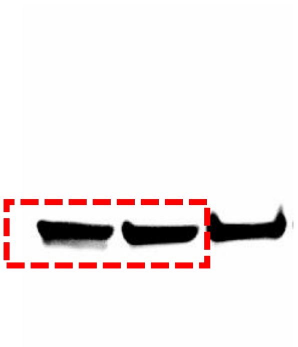

exosome-GAPDH

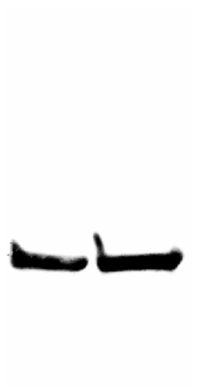

Figure S4A

Foxp1

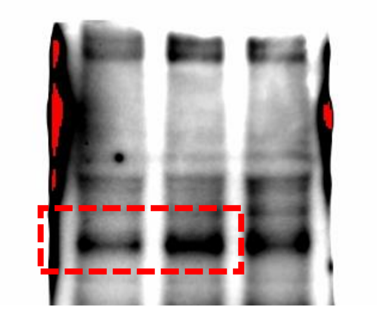

GAPDH

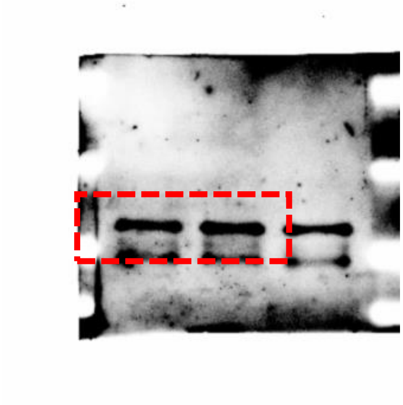

Figure S5E

CD63

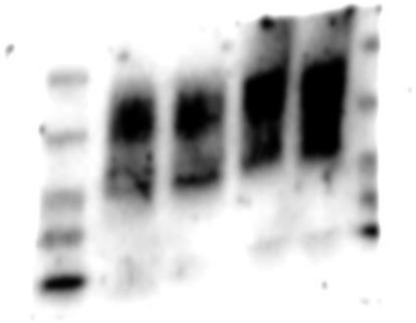

TSG101

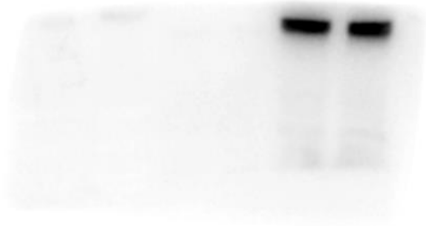

Alix

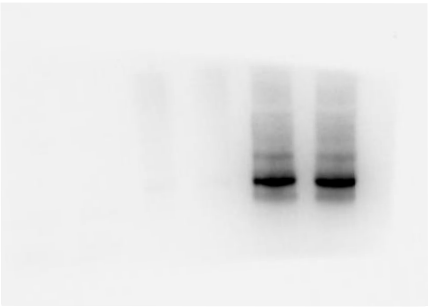

Calnexin

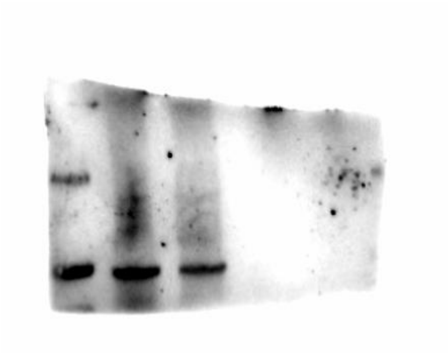

Supplement: Supplementary file 2 — Supporting Information [file ADVS-12-e15712-s001.zip › WB_Unprocessed_Images.pdf]
